# Association of Economic Status and Educational Attainment With Posttraumatic Stress Disorder: A Mendelian Randomization Study

**DOI:** 10.1001/jamanetworkopen.2019.3447

**Published:** 2019-05-03

**Authors:** Renato Polimanti, Andrew Ratanatharathorn, Adam X. Maihofer, Karmel W. Choi, Murray B. Stein, Rajendra A. Morey, Mark W. Logue, Caroline M. Nievergelt, Dan J. Stein, Karestan C. Koenen, Joel Gelernter

**Affiliations:** 1 Department of Psychiatry, Yale University School of Medicine, West Haven, Connecticut; 2 Veterans Affairs Connecticut Healthcare Center, West Haven, Connecticut; 3 Department of Epidemiology, Mailman School of Public Health, Columbia University, New York, New York; 4 Department of Psychiatry, School of Medicine, University of California, San Diego; 5 Veterans Affairs Center of Excellence for Stress and Mental Health and Research Service, Veterans Affairs San Diego Healthcare System, San Diego, California; 6 Stanley Center for Psychiatric Research, Broad Institute, Cambridge, Massachusetts; 7 Department of Psychiatry, Massachusetts General Hospital, Boston; 8 Department of Epidemiology, Harvard T.H. Chan School of Public Health, Harvard University, Boston, Massachusetts; 9 Psychiatry Service, Veterans Affairs San Diego Healthcare System, San Diego, California; 10 Department of Psychiatry, Duke University, Durham, North Carolina; 11 Durham Veterans Affairs Medical Center, Durham, North Carolina; 12 National Center for PTSD Behavioral Science Division, Veterans Affairs Boston Healthcare System, Boston, Massachusetts; 13 Department of Psychiatry, School of Medicine, Boston University, Boston, Massachusetts; 14 South African Medical Research Council Unit on Risk and Resilience in Mental Disorders, Department of Psychiatry, University of Cape Town, Cape Town, South Africa; 15 Department of Genetics, School of Medicine, Yale University, New Haven, Connecticut; 16 Department of Neuroscience, School of Medicine, Yale University, New Haven, Connecticut

## Abstract

**Importance:**

There is a well-established negative association of educational attainment (EA) and other traits related to cognitive ability with posttraumatic stress disorder (PTSD), but the underlying mechanisms are poorly understood.

**Objectives:**

To investigate the association of PTSD with traits related to EA.

**Design, Setting, and Participants:**

Genetic correlation, polygenic risk scoring, and mendelian randomization (MR) were conducted including 23 185 individuals with PTSD and 151 309 control participants from the Psychiatric Genomics Consortium for PTSD and up to 1 131 881 individuals assessed for EA and related traits from UK Biobank, 23andMe, and the Social Science Genetic Association Consortium. Data were analyzed from July 3 through November 19, 2018.

**Main Outcomes and Measures:**

Genetic correlation obtained from linkage disequilibrium score regression, phenotypic variance explained by polygenic risk scores, and association estimates from MR.

**Results:**

Summary association data from multiple genome-wide association studies were available for a total of 1 180 352 participants (634 391 [53.7%] women). Posttraumatic stress disorder showed negative genetic correlations with EA (*r*_g_ = −0.26; SE = 0.05; *P* = 4.60 × 10^−8^). Mendelian randomization analysis, conducting considering a random-effects inverse-variance weighted method, indicated that EA has a negative association with PTSD (β = −0.23; 95% CI, −0.07 to −0.39; *P* = .004). Investigating potential mediators of the EA-PTSD association, propensity for trauma exposure and risk-taking behaviors were observed as risk factors for PTSD independent of EA (trauma exposure: β = 0.37; 95% CI, 0.19 to 0.52; *P* = 2.57 × 10^−5^; risk-taking: β = 0.76; 95% CI, 0.38 to 1.13; *P* = 1.13 × 10^−4^), while income may mediate the association of EA with PSTD (MR income: β = −0.18; 95% CI, −0.29 to −0.07; *P* = .001; MR EA: β = −0.23; 95% CI, −0.39 to −0.07; *P* = .004; multivariable MR income: β = −0.32; 95% CI, −0.57 to 0.07; *P* = .02; multivariable MR EA: β = −0.04; 95% CI, −0.29 to 0.21; SE, 0.13; *P* = .79).

**Conclusions and Relevance:**

Large-scale genomic data sets add further evidence to the negative association of EA with PTSD, also supporting the role of economic status as a mediator in the association observed.

## Key Points

**Question:**

Which mechanisms underlie the negative association of posttraumatic stress disorder (PTSD) with traits related to educational attainment (EA)?

**Findings:**

In this mendelian randomization study based on large-scale genomic data sets, including data from more than 1 million individuals, EA was negatively associated with PTSD, also supporting the role of economic status as a mediator in this association.

**Meaning:**

This study suggests that economic status may mediate the association of EA with PTSD independent of the brain mechanisms associated with EA.

## Teaser

This mendelian randomization study investigates the association of posttraumatic stress disorder and traits associated with educational attainment.

## Introduction

Posttraumatic stress disorder (PTSD) is a psychological condition that occurs in some individuals after exposure to a major traumatic event. Prospective studies have suggested that many variables previously considered outcomes of trauma are likely to be pretrauma risk factors.^1^ Among these complex associations, that of PTSD with cognitive ability and educational attainment (EA; ie, the number of years of schooling that individuals complete) is among the most puzzling.

While there is a robust epidemiologic literature on the negative association of PTSD with cognitive ability and EA,^2,3,4,5^ the underlying mechanisms remain unclear. Reverse causation (ie, when an outcome precedes and causes the exposure)^6,7,8^ is a key obstacle to disentangling the direction of the mechanisms that associate these 2 phenotypes.

Adequately powered genome-wide association studies (GWASs) are able to dissect the predisposition to complex traits. This requires extremely large sample sizes to detect the polygenic architecture of complex traits, overcoming their heterogeneity to have sufficient power to find risk loci of very small effect.^9^ By combining such small-effect loci, it is possible to build genetic instruments that can be used to investigate complex epidemiological associations, such as the underlying mechanisms connecting PTSD, traits related to EA, and the potential mediation of other pretrauma risk factors. In particular, genetic information can remove the bias of reverse causation from analysis of the association of PTSD with cognition and education. Genetic variants are allocated at conception and do not change throughout life, and they can be used to define reliable genetic instruments that can be applied in a mendelian randomization (MR) analysis.^10,11,12,13,14^ The basic principle in MR is that an instrumental variable based on genetic variants associated with a phenotype can be used to represent, or mirror, the disease risk associated with that phenotype without the presence of possible environmental confounders.^15^

Previous large-scale GWASs conducted by the Social Science Genetic Association Consortium (SSGAC)^16,17,18,19^ investigated traits related to EA, identifying a number of loci and biological pathways regulating brain mechanisms at the basis of human cognitive ability. A 2018 genome-wide analysis of multiple brain disorders and phenotypes^20^ showed pervasive shared heritability of these traits with EA and related traits. Based on the GWAS regarding PTSD newly generated by the Psychiatric Genomics Consortium (PGC),^21^ we applied multiple statistical methods to large-scale genomic data sets to investigate the mechanisms involved in the association of PTSD with traits related to EA and potential pretrauma risk factors.

## Methods

This study was conducted using summary association data generated by previous studies. Owing to the use of previously collected, deidentified, aggregated data, this study did not require institutional review board approval. Ethical approval had been obtained in all original studies.^16,21^ Summary data were available for a total of 1 180 352 participants. Multiple statistical methods were applied to these data sets to investigate the association of PTSD with EA and related traits. A schematic workflow summarizing the analyses conducted is reported in eFigure 1 in the Supplement. These analyses were conducted from July 3 through November 19, 2018. The study was reported in accordance to the Strengthening the Reporting of Genetic Association Studies (STREGA) reporting guideline.^22^

### Cohorts Investigated

Genome-wide information regarding PTSD was derived from the freeze-2 analysis conducted by the Psychiatric Genomics Consortium Posttraumatic Stress Disorder (PGC-PTSD) Working Group.^21^ In this analysis, lifetime and/or current PTSD status was assessed using various instruments and different versions of the *Diagnostic and Statistical Manual of Mental Disorders* (Third Edition Revised, Fourth Edition, and Fifth Edition). We focused on the data generated from the analysis of individuals of European descent (23 185 individuals with PTSD; 151 309 control participants) because the genome-wide analyses of traits associated with EA were conducted only on this ancestry group.

Genome-wide information regarding traits related to EA were derived from the GWAS meta-analysis by the SSGAC,^16^ which investigated EA as the primary phenotype in a total of 1 131 881 individuals (EA1M). Additional information about the definition of the EA phenotype is available in the eAppendix in the Supplement. In the same SSGAC study,^16^ 3 additional phenotypes were investigated. Of these, 2 were analyzed exclusively among research participants of the personal genomics company 23andMe. Participants were asked to rate their mathematical ability (MA; n = 564 698; very poor, 0; poor, 1; about average, 2; good, 3; excellent, 4) and to list the most advanced math course they had successfully completed (MC; n = 430 445; prealgebra, 1; algebra, 2; geometry, 3; trigonometry, 4; precalculus, 5; calculus, 6; vector calculus, 7; >vector calculus, 8). The third phenotype investigated, cognitive performance, was assessed in 257 828 participants from the Cognitive Genomics Consortium study and the UK Biobank.^16^ For the data sets including participants from 23andMe, we had access only to summary association data of the top 10 000 variants. Accordingly, the data sets derived from 23andMe were not used for the reverse analysis (ie, estimating the association of PTSD with traits related to EA).

A summary of the data sets tested is reported in the Table. Since UK Biobank participants were included in both PGC-PTSD and SSGAC studies, some analyses were conducted on a PGC-PTSD subsample that excluded the UK Biobank cohort (PGC-PTSD freeze-1.5 data set: 12 823 individuals with PTSD; 35 648 control participants). Excluding UK Biobank, negligible overlap is present between PGC-PTSD and SSGAC cohorts (eAppendix in the Supplement).

**Table.** Traits Tested With Corresponding Information Regarding Sample Size, Data Available, and Cohorts Included

Trait	Abbreviation	Sample Size	Cohort	Summary Statistics
Posttraumatic stress disorder	PTSD freeze-1.5	12 823 with PTSD; 35 648 controls	PGC	Full
Posttraumatic stress disorder	PTSD freeze-2	23 185 with PTSD; 151 309 controls	PGC, UKB	Full
Cognitive performance	CP	257 828	SSGAC, UKB	Full
Educational attainment	EA	766 345	SSGAC, UKB	Full
Educational attainment	EA1M	1 131 881	SSGAC, UKB, 23andMe	Top 10 000
Self-reported math ability	MA	564 692	23andMe	Top 10 000
Most advanced math course completed	MC	430 439	23andMe	Top 10 000
Risk-taking behaviors	RT	348 549	UKB	Full
Income	Income	311 028	UKB	Full
Physically abused by family as a child	PAC	117 838	UKB	Full

Abbreviations: PGC, Psychiatric Genomics Consortium; PTSD, posttraumatic stress disorder; SSGAC, Social Science Genetic Association Consortium; UKB, UK Biobank.

We also used data from the UK Biobank to investigate the possible mediation of PTSD risk factors in their associations with traits related to EA. Self-reported risk-taking behaviors were assessed with the question, “Would you describe yourself as someone who takes risks?” (UK Biobank data field 2040). Income was assessed with, “Average total household income before tax” (UK Biobank data field 738). We also considered traumatic events assessed in the UK Biobank (eTable 1 in the Supplement). Genome-wide information regarding these traits was derived from GWAS summary association data generated from the UK Biobank.^23^

### Genetic Correlation and Definition of the Genetic Instruments

Linkage disequilibrium (LD) score regression was used to estimate the genetic correlation among the traits investigated.^24^ Since sample overlap between the GWAS tested does not affect the results obtained from this method,^24^ we were able to calculate pairwise genetic correlations, including those between data sets that had overlapping information with the UK Biobank participants.

The polygenic risk scores (PRSs) were calculated after using *P* value–informed clumping with an LD cutoff of *R*^2^ = 0.001 within a 10 000-kilobase window, excluding the major histocompatibility complex region of the genome because of its complex LD structure and including only variants with a minor allele frequency less than 1%. The European samples from the 1000 Genomes Project were used as the LD reference panel.^25^ The PRS analysis was conducted on the basis of the GWAS summary association data using the gtx R package incorporated in PRSice software.^26^ For each PRS analysis, we calculated an approximate estimate of the explained variance from a multivariate regression model.^27^ For the traits related to EA, we considered a genome-wide significance threshold (*P* < 5.00 × 10^−8^). For other traits (ie, PTSD, income, risk-taking behaviors, and trauma exposure), owing to the limited power to detect a large number of genome-wide significant loci, we evaluated multiple *P* value thresholds (PT; PT = 5.00 × 10^−8^, 10^−7^, 10^−6^, 10^−5^, 10^−4^, .001, .05, .1, .3, .5, and PT < 1) to increase the variance explained by the genetic instruments. To account for the multiple PTs tested, we considered *P* < .005 as the significance threshold in the PRS analysis. Tests of statistical significance were 2-tailed. The results of the PRS analyses were used to define the genetic instruments for each pairwise comparison to be investigated further via the MR approach.

### Mendelian Randomization

To assess the association among the traits tested, we used GWAS summary association data to conduct 2-sample MR analyses.^28^ As mentioned earlier, genetic instruments were based on the results obtained in the PRS analysis. Since different MR methods have different sensitivities to different potential issues, accommodate different scenarios, and vary in their statistical efficiency,^11^ we considered a range of MR methods. The primary analysis was conducted considering a random-effects inverse-variance weighted (IVW) method.^29^ The secondary MR methods included MR Egger,^30^ simple mode,^31^ weighted median,^32^ and weighted mode.^31^ These MR analyses were conducted using the TwoSampleMR R package.^29^ Additionally, owing to the fact that some traits showed a limited number of associated genome-wide significant variants, genetic instruments associated with PTSD, income, risk-taking behaviors, and trauma exposure were based on suggestive PTs similar to previous MR studies.^33,34,35^ We verified these IVW estimates using the MR–Robust Adjusted Profile Score (MR-RAPS) approach, which is a method designed to identify and estimate confounded associations using weak genetic instrument variables.^36,37^ We conducted multiple sensitivity analyses with respect to the MR tests conducted to exclude possible biases (horizontal pleiotropy, ie, the variants included in the genetic instrument having an effect on disease outside their effects on the exposure in MR^38,39^) under different scenarios in the MR estimates. These included the IVW heterogeneity test,^29^ the MR-Egger intercept,^30^ the MR-RAPS overdispersion test,^36^ and the MR–Pleiotropy Residual Sum and Outlier (MR-PRESSO) global test.^40^ Finally, a leave-1-out analysis was conducted to identify potential outliers among the variants included in the genetic instruments tested. The MR results without evidence of horizontal pleiotropy and heterogeneity were entered in the multivariable MR (MVMR) analysis^41^ conducted using the IVW approach. This method permits evaluation of the independent association of each risk factor with the outcome, similar to the simultaneous assessment of several treatments in a factorial randomized trial.^41^

Comparing the utility of MR with MVMR methods, MR estimates the total association of the exposure with the outcome, whereas MVMR estimates the direct association of each exposure with the outcome.^42^ In this scenario, MVMR is not a form of mediation analysis but instead estimates the direct association of the exposure with the outcome that does not act via the mediator.^42^ The MVMR analysis was conducted using the MendelianRandomization R package.^43^

### Enrichment Analysis

We tested for functional differences between the traits of interest by means of enrichment analyses based on tissue-specific and cell type–specific gene expression reference panels.^44,45,46,47,48^ These analyses were conducted using the MAGMA tool^49^ implemented in FUMA.^50^

## Results

Data were available for a total of 1 180 352 participants (634 391 [53.7%] women). Information regarding PTSD was available for 23 185 individuals with PTSD and 151 309 control participants (174 494 individuals; 15%) from the PGC-PSTD Working Group and regarding EA for 1 131 881 individuals (96%) from the total sample.

### Genetic Correlation and Polygenic Risk Scoring

Our study investigated multiple data sets generated from different cohorts and with different data availability (Table). Using LD score regression and data sets with full GWAS summary association data, we observed a negative genetic correlation of PTSD (freeze-2) with EA (*r*_g_ = −0.26; SE = 0.05; *P* = 4.60 × 10^−8^) and cognitive performance (*r*_g_ = −0.16; SE = 0.05; *P* = 9.00 × 10^−4^). The PRS analyses were conducted using PTSD as the target and traits related to EA as the training data set, considering only genome-wide significant variants (*P* < 5.00 × 10^−8^; eFigure 2 in the Supplement). This analysis was conducted excluding those pairwise comparisons that would have included data sets with the UK Biobank as an overlapping cohort. The most significant PRS association was observed between MC genome-wide significant PRS with respect to PGC-PTSD freeze-2 data (*R*^2^ = 0.04%; SE = 0.0001; *P* = 1.13 × 10^−8^). The same PTSD data set showed a weaker association with MA PRS (*R*^2^ = 0.01%; SE =0.00003; *P* = .004). Significant associations were also observed with respect to PGC-PTSD freeze-1.5 outcome for EA (*R*^2^ = 0.03%; SE = 0.0001; *P* = 6.16 × 10^−4^), MC PRS (*R*^2^ = 0.03%; SE = 0.0001; *P* = 7.86 × 10^−4^), and EA1M PRS (*R*^2^ = 0.03%; SE = 0.0001; *P* = .002). The phenotypic variance explained by the significant PRS is in line with the cross-phenotype association expected between 2 complex traits with a moderate genetic correlation. No association of the PRS of cognitive performance and MA was observed with respect to PGC-PTSD freeze-1.5 data set (cognitive performance: *R*^2^ < 0.01%; *P* = .15; MA: *R*^2^ < 0.01%; *P* = .11), and accordingly, these phenotypes were not investigated further. We tested the reverse direction (ie, PTSD as base and traits related to EA as target). Owing to the limited number of genome-wide significant loci in the PGC-PTSD analysis, the PRS analysis was conducted considering multiple-association PTs to include at least 10 LD-independent variants in each PRS tested (eFigure 3 in the Supplement). We observed a nominally significant association between the PGC-PTSD freeze-1.5 PRS (PT = 10^−5^) and EA (*R*^2^ = 0.0004%; SE = 0.000002; *P* = .04) that would not survive a Bonferroni correction for the number of PTs tested.

### Mendelian Randomization

**Figure 1.**
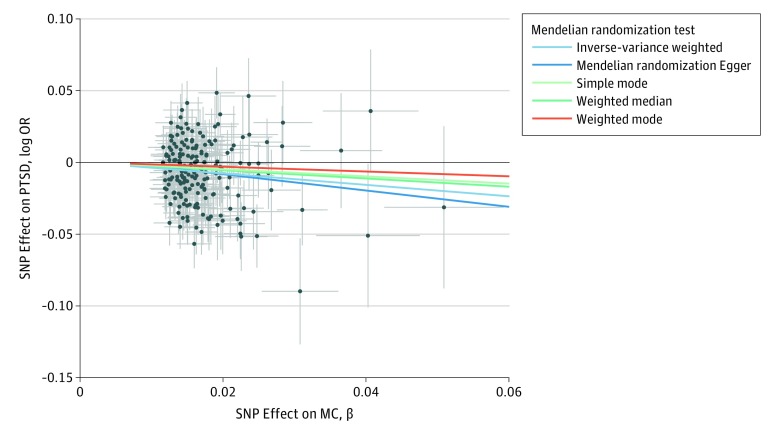
Single-Nucleotide Polymorphism (SNP) Repeated Effects on Posttraummatic Stress Disorder (PTSD) and Most Advanced Math Course Completed (MC) SNP exposure (MC associations, β) and SNP outcome (PTSD freeze-2 associations, log odds ratio [OR]) coefficients used in the mendelian randomization analysis. Crosses represent 95% CIs for each association.

**Figure 2.**
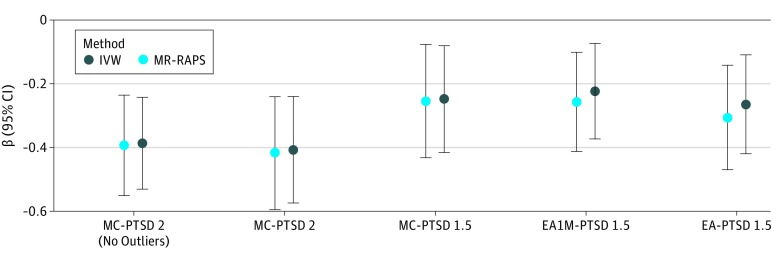
Estimated Associations Considering Different Traits Associated With Educational Attainment (EA) and 2 Versions of the Posttraumatic Stress Disorder (PTSD) Data Set EA1M indicates sample including participants from Social Science Genetic Association Consortium, UK Biobank, and 23andMe; IVW, inverse-variance weighted; MC, most advanced math course completed; MR-RAPS, mendelian randomization–robust adjusted profile score; PTSD2, PTSD freeze 2 data set; and PTSD1.5, PTSD freeze 1.5 data set.

Based on the PRS results, we conducted MR tests using 3 genetic instruments based on genome-wide significant variants. These included MC, EA, and EA1M, tested with respect to PGC-PTSD data sets (freeze-2 and/or freeze-1.5, depending on UK Biobank overlap). The reverse MR test was conducted on the basis of PGC-PTSD freeze-1.5 data (PT =  5.00 × 10^−5^) with respect to the EA data set. It was not possible to conduct reverse analyses for the MC and EA1M data sets because we did not have access to the full GWAS summary association data for these studies. A significant association was found between MC and PGC-PTSD freeze-2 data (IVW: β = −0.41; 95% CI, −0.59 to −0.23; *P* = 3.46 × 10^−6^). Concordant results were observed when considering other MR methods (Figure 1). The significance was replicated with the MR-RAPS approach (β = −0.42; 95% CI, −0.60 to −0.24; SE = 0.09; *P* = 3.70 × 10^−6^), and no outliers were identified by the leave-1-out analysis (eFigure 4 in the Supplement). However, we observed the presence of possible bias in this result owing to heterogeneity and/or pleiotropy (IVW heterogeneity test: *Q* = 267.4; *df* = 192; *P* = 2.61 × 10^−4^; MR-RAPS overdispersion test: estimated pleiotropy variance, 0.0001; *P* = .007; MR-PRESSO global test: observed residual sum of squares, 281.4; *P* = 5.00 × 10^−4^). Thus, we identified the outliers on the basis of the MR-RAPS standardized residuals (−1.96 > *z* > 1.96) and verified their contributions on the results of the IVW heterogeneity test (eFigure 5 in the Supplement). Removing the outliers from the MC genetic instrument, we confirmed the association of MC with PTSD in the freeze-2 data set (IVW: β = −0.39; 95% CI, −0.57 to 0.21; *P* = 4.25 × 10^−7^; MR-RAPS: β = −0.39; 95% CI, −0.55 to −0.23; *P* = 1.06 × 10^−6^) and the lack of evidence of possible confounders from the sensitivity analyses (eTable 2 and eFigure 6 in the Supplement). We verified the reliability of this MR finding considering PGC-PTSD freeze-1.5 data as the outcome and MC, EA, and EA1M as exposures. We observed comparable MR results across the genetic instruments generated from different cohorts and the 2 versions of the PGC-PTSD data sets (Figure 2; eFigure 7 in the Supplement). However, consistent with the lower power of PGC-PTSD freeze-1.5 data, we observed a reduction of the significance (MC for PGC-PTSD freeze-2 data set: IVW: β = −0.41; 95% CI, −0.59 to −0.23; *P* = 3.46 × 10^−6^; MC for PGC-PTSD freeze-1.5 data set: IVW: β = −0.22; 95% CI, −0.38 to −0.06; *P* = .004; EA for PGC-PTSD freeze-1.5 data set: β = −0.23; 95% CI, −0.39 to −0.07; *P* = .004). We verified that no bias in the MR results was present owing to palindromic variants with an ambiguous allele frequency^51^ and to the presence of assortative mating in EA^52^ (eTable 3 and eAppendix in the Supplement).

To support further that MC and EA are associated with the same mechanism, we conducted a MVMR analysis, which showed that these 2 associations are not independent from each other (eFigure 8 in the Supplement), and their relationship with PTSD should be shared. Accordingly, we used EA as a proxy of MC in the subsequent analyses because we had full access only to the former data set.

We tested the reverse association, considering PTSD as the risk factor (ie, exposure) and EA as the outcome of the MR analysis. We included PTSD genetic instrument variants with a PTSD GWAS *P* = 10^−5^ considering the PGC-PTSD freeze-1.5 data. No significant association was observed (IVW: β = 0.006; 95% CI, −0.002 to 0.014; *P* = .16; MR-RAPS: β = 0.0006; 95% CI, −0.007 to 0.008; *P* = .10). To further confirm the absence of reverse association, we conducted an MR analysis including all LD-independent variants in the genetic instrument and applied the MR-RAPS method only. No directional association of PTSD with EA was observed (β = −0.0006; 95% CI, −0.002 to 0.002; *P* = .55), but we confirmed a directional association of EA with PSTD (β = −0.27; 95% CI, −0.38 to −0.15; *P* = 8.06 × 10^−6^). These outcomes were stable across different adjustments of the MR-RAPS method (eTable 4 in the Supplement).

### Multivariable MR Analysis

To further investigate the association of EA with PTSD, we tested 3 potential mediators (risk-taking behaviors, income, and trauma exposure) in an MVMR analysis. Before entering these potential mediators in the MVMR analysis, we verified the reliability of each genetic instrument by conducting a standard 2-sample MR and verifying the evidence of bias owing to heterogeneity and horizontal pleiotropy. Because of the limited number of genome-wide significant variants with respect to these traits, we conducted a PRS considering multiple PTs as described earlier, to determine the best genetic instrument for each trait with respect to the PGC-PTSD freeze-1.5 data set (eFigure 9 in the Supplement). The best results were observed for PT = 5.00 × 10^−4^ with risk-taking behaviors (*R*^2^ = 0.06%; SE = 0.0001; *P* = 1.53 × 10^−5^) and PT = .001 for income (*R*^2^ = 0.09%; SE = 0.0002; *P* = 5.67 × 10^−8^). The MR analysis based on these genetic instruments confirmed that PTSD is associated with the genetic instruments related to risk-taking behaviors (IVW: β = 0.76; 95% CI, 0.38 to 1.13; *P* = 1.13 × 10^−4^; MR-RAPS: β = 0.76; 95% CI, 0.32 to 1.21; *P* = 6.75 × 10^−4^), and income (IVW: β = −0.18; 95% CI, −0.29 to −0.07; *P* = .001; MR-RAPS: β = −0.19; 95% CI, −0.31 to −0.07; *P* = .003). No evidence of heterogeneity or pleiotropy was observed in either analysis (eTable 5 in the Supplement).

Multiple traumatic events were assessed in the UK Biobank (eTable 1 in the Supplement), and they showed genetic correlation with each other (eTable 6 in the Supplement). We selected 4 traumatic experiences that showed a similar pattern of genetic correlation with respect to PTSD, EA, and the other 2 potential mediators (eFigure 10 in the Supplement). The most informative PRS across the 4 traumatic experiences tested was observed at PT = .001 (eFigure 11 in the Supplement). Then, we conducted an MR analysis testing different trauma-related genetic instruments with respect to PGC-PTSD freeze-1.5 data. We observed significant associations not affected by confounders (eTable 7 in the Supplement) for 3 traumatic experiences and, conducting an MVMR analysis, identified “physically abused by family as a child” (UK Biobank data field 20488) as the most informative genetic instrument for trauma exposure (MR analysis: β = 0.36; 95% CI, 0.19 to 0.52; *P* = 2.57 × 10^−5^; MVMR analysis: β = 0.26; 95% CI, −0.51 to 0.01; SE = 0.129; *P* = .04) (eFigure 12 in the Supplement).

**Figure 3.**
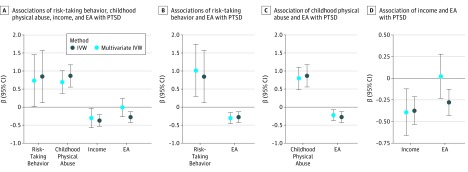
Multivariable Mendelian Randomization Analysis Considering Associations of Educational Attainment (EA), Posttraumatic Stress Disorder (PTSD), and Other Traits IVW indicates inverse-variance weighted.

In the MVMR analysis, we observed that trauma exposure and risk-taking behaviors were independent risk factors for PTSD (ie, the results obtained from the MR IVW and MVMR IVW analyses were both significant; Figures 3A, B, and C). Conversely, the genetic instrument related to income potentially mediates the association of the EA genetic instrument with PTSD (Figure 3A and D). Educational attainment has a significant association with respect to PTSD (β = −0.23; 95% CI, −0.39 to −0.07; *P* = .004), but when adjusted by income, this result is null (β = −0.04; 95% CI −0.30 to 0.21; *P* = .79). Conversely, the association of income with PTSD was still significant when adjusted by EA (unadjusted: β = −0.18; 95% CI, −0.30 to −0.06; *P* = .001; adjusted: β = −0.32; 95% CI, −0.57 to −0.07; SE = 0.13; *P* = .02).

### Enrichment Analysis

**Figure 4.**
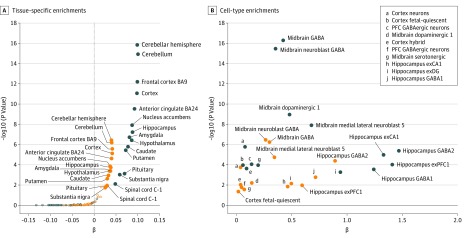
Enrichment Analyses for Educational Attainment and Income A, Tissue-specific enrichments (brain tissues are indicated with solid circles; nonbrain tissues are indicated with open circles). B, Cell-type enrichments that were at least nominally significant (*P* < .05) in both traits. BA9 indicates Brodmann area 9; BA24, Brodmann area 24; exCA1, excitatory neurons in hippocampal subfield CA1; exDG, excitatory neurons in dentate gyrus; exPFC1, excitatory neurons in prefrontal cortex 1; GABA, γ-aminobutyric acid; and PFC, prefrontal cortex.

Although there is a large genetic correlation between EA and income (*r* = 0.81; *P* < 6.10 × 10^−308^), income is significantly more correlated with PTSD than EA (EA: *r* = −0.26; SE = 0.05; *P* = 4.60 × 10^−8^; income: *r* = −0.45; SE = 0.06; *P* = 9.98 × 10^−16^; *z *for EA × income = 2.65; *P *for EA × income = 0.008). Both traits are enriched for the transcriptomic profile of multiple brain tissues (eg, cerebellar hemisphere, EA: β = 0.01; 95% CI, 0.07-0.12; *P* = 1.49 × 10^−16^; income: β = 0.04; 95% CI, 0.02-0.06; *P* = 3.83 × 10^−7^) and neuronal cell types (eg, γ-aminobutyric acid [GABA]–ergic neurons, EA: β = 0.14; 95% CI, 0.07-0.21; *P* = 8.96 × 10^−5^; income: β = 0.05; 95% CI, 0.01-0.09; *P* = .02), but the enrichment signals of the EA GWAS are more significant than the ones observed in the income GWAS (Figure 4; eTable 8 in the Supplement), showing that EA data are more informative for the brain processes expected to be associated with cognition than income data.

## Discussion

Our analysis was based mainly on data from investigations of traits associated with EA. Previous studies have demonstrated that genetic results deriving from them are mainly informative regarding the brain mechanisms at the basis of human cognitive ability.^16,17,18,19^ Our analysis made use of these data to show that traits associated with cognitive ability have an association with PTSD. This result was consistent across traits in independent cohorts, even when adjusting the analysis for assortative mating present in these traits.^52^ In line with an association direction from cognition to PTSD, no evidence of reverse association was observed.

Although our findings are consistent with a specific direction, we had to evaluate whether other factors could be responsible for this association. Accordingly, we tested 3 phenotypes of known relevance for PTSD: propensity to trauma exposure,^53^ risk-taking behaviors,^54^ and economic status.^55^ Our MVMR analysis clearly showed that, while propensity to trauma exposure and risk-taking behaviors are independent PTSD risk factors, the directional association of EA with PTSD is associated with economic status, which is the driving force of the association. Educational attainment and income showed a large genetic overlap, and while PTSD showed a higher correlation with income than with EA, our investigation showed that EA is more informative for the brain mechanisms that are considered responsible for a predisposition to high cognitive ability. Since income appears to be responsible for the EA-PTSD association, we hypothesize that this mechanism is not related to cognitive ability but rather to other risk factors. This is also supported by our finding that, unlike EA and MC (traits that should be more associated with socioeconomic status), cognitive performance and MA showed a lower genetic association with PTSD. A 2018 genome-wide investigation of social stratification^56^ showed that cognition and socioeconomic status are correlated with a wide range of factors, including personality, psychological traits, mental health, substance use, physical health, reproductive behaviors, and anthropometric traits. Additionally, income may reflect indirect effects, such as those induced by genetic nurture on EA.^57,58^ Socioeconomic factors might also be associated with the outcome of PTSD, given that individuals with poorer outcomes are more likely to be included in studies of prevalent cases.

To our knowledge, this study represents the first MR analysis to investigate the underlying mechanisms linking cognitive ability to PTSD. It is based on the largest genome-wide data sets for these traits available at this time. The EA-PTSD association observed is in line with several prospective studies,^1^ and the association of socioeconomic status with PTSD is also a well-established risk factor reported in several observational studies.^55,59^ Compared with these previous investigations, the current analyses are based on a much larger population (>1 million individuals) than would ever be feasible for a traditional experimental design (which would include randomized interventions and measurements of outcomes) and without the ethical quandaries that would accompany such randomizations. Also, these results are not expected to be affected by reverse association because of the genetic information used. The findings show that income may explain the EA-PTSD association, suggesting that brain mechanisms related to cognitive ability are not directly responsible for the association observed.

### Limitations

Our study has limitations. The present study is based on genetic information generated from the investigation of heterogeneous, clinically defined phenotypes such as PTSD. We tested this complex trait with respect to a series of complex, socially contextualized phenotypes, including EA, income, risk-taking behaviors, and predisposition to traumatic events. Although we used appropriate statistical methods and conducted the analyses across multiple independent cohorts, findings related to genetic data associated with these phenotypes need to be interpreted cautiously.^58,60^ The results of our current analysis are also limited by the statistical power of the PTSD GWAS data sets, which may have limited our ability to observe the reverse association of PTSD with EA. However, we used multiple methods that showed significant associations when applied to similarly powered GWAS data sets. Another potential limitation is owing to the pervasive presence of horizontal pleiotropy among complex traits.^39^ We applied multiple sensitivity analyses that accounted for different scenarios related to the potential confounding effect of horizontal pleiotropy and heterogeneity in the genetic instruments applied in our MR analyses. Although no evidence of bias was observed by the methods used, our current findings could be affected by an unaccounted confounder.

## Conclusions

This study provides new evidence to elucidate the association of EA with PTSD, pointing toward risk factors associated with economic status rather than brain pathways. These findings have relevant implications with respect to our understanding of the pretrauma risk factors associated with increased vulnerability to PTSD. Additionally, MR analysis should consider testing the independence of multiple correlated risk factors with respect to the outcome of interest. This is particularly relevant when investigating the potential role of EA in human phenotypes and disorders.

## Notes

Supplement. **eAppendix. **Phenotype Definitions, Sample Overlap, Palindromic Variants, and Assortative Mating **eReferences.** **eTable 1.** Traumatic Experiences Assessed in the UK Biobank **eTable 2.** Results of the Sensitivity Analyses Conducted With Respect to the MathClass→PTSD Test With and Without the Outlier Variants in the MathClass Genetic Instrument **eTable 3.** Results of the IVW Analyses Considering Genetic Instruments With and Without Palindromic Variants With Ambiguous Allele Frequencies (PAL and noPAL, Respectively) **eTable 4.** MR-RAPS Analysis Considering Various Adjustments Based on Genome-Wide Genetic Instruments **eTable 5.** Results of the Sensitivity Analyses Conducted With Respect to the Income→PTSD and Risk-Tak→PTSD Tests **eTable 6.** Genetic Correlation Among Trauma Experiences Assessed in the UK Biobank **eTable 7.** Results (Causal Effects and Sensitivity Analyses) of the MR Test Conducted Using Trauma-Related Genetic Instruments With Respect to PTSD **eTable 8.** Results of the Enrichment Analysis Based on Tissue-Specific and Cell Type–Specific Transcriptomic Data **eFigure 1. **Schematic Workflow of the Analyses Conducted **eFigure 2.** Genetic Correlations Estimated Between Traits Related to Cognitive Ability and 2 Versions of the Posttraumatic Stress Disorder Data Set, PGC-PTSD Freeze-2 (2) and PGC-PTSD Freeze-1.5 (1.5) **eFigure 3.** Effect of the PTSD PRS on Educational Attainment (Yellow) and Cognitive Performance (Green) Considering Different Inclusion Thresholds **eFigure 4.** Leave-1-Out Analysis Conducted With Respect to the MathClass→PTSD2 Result **eFigure 5.** Identification of Potential Outliers (in Red) in MathClass Genetic Instrument Based on IVW Heterogeneity Test and MR-RAPS Standardized Residuals **eFigure 6.** Results of the MathClass→PTSD2 Analysis After the Removal of the Potential Outliers From the Genetic Instrument **eFigure 7.** Results of the Sensitivity Analyses With Respect to All MR Analyses Conducted **eFigure 8.** Multivariable Mendelian Randomization Analysis Considering the Effects of EdAtt and MathClass on PTSD **eFigure 9.** Effect of Income and RiskTak PRS (Green and Blue, Respectively) on PTSD Considering Different Inclusion Thresholds **eFigure 10.** Genetic Correlations Between Traumatic Experiences and the Other Traits of Interest **eFigure 11.** Effect of Trauma-Related PRS on PTSD Considering Different Inclusion Thresholds **eFigure 12.** Multivariable Mendelian Randomization Analysis Considering the Effects of Physically Abused by Family as a Child and Belittlement by Partner or Ex-Partner as an Adult on PTSD
