# Supplementary material for: Association of Economic Status and Educational Attainment With Posttraumatic Stress Disorder: A Mendelian Randomization Study
Source: JAMA Netw Open. 2019 May 3;2(5):e193447. doi: 10.1001/jamanetworkopen.2019.3447 (PMC6503495; doi:10.1001/jamanetworkopen.2019.3447)
Supplement: Supplement. — eAppendix. Phenotype Definitions, Sample Overlap, Palindromic Variants, and Assortative Mating eReferences. eTable 1. Traumatic Experiences Assessed in the UK Biobank eTable 2. Results of the Sensitivity Analyses Conducted With Respect to the MathClass→PTSD Test With and Without the Outlier Variants in the MathClass Genetic Instrument eTable 3. Results of the IVW Analyses Considering Genetic Instruments With and Without Palindromic Variants With Ambiguous Allele Frequencies (PAL and noPAL, Respectively) eTable 4. MR-RAPS Analysis Considering Various Adjustments Based on Genome-Wide Genetic Instruments eTable 5. Results of the Sensitivity Analyses Conducted With Respect to the Income→PTSD and Risk-Tak→PTSD Tests eTable 6. Genetic Correlation Among Trauma Experiences Assessed in the UK Biobank eTable 7. Results (Causal Effects and Sensitivity Analyses) of the MR Test Conducted Using Trauma-Related Genetic Instruments With Respect to PTSD eTable 8. Results of the Enrichment Analysis Based on Tissue-Specific and Cell Type–Specific Transcriptomic Data eFigure 1. Schematic Workflow of the Analyses Conducted eFigure 2. Genetic Correlations Estimated Between Traits Related to Cognitive Ability and 2 Versions of the Posttraumatic Stress Disorder Data Set, PGC-PTSD Freeze-2 (2) and PGC-PTSD Freeze-1.5 (1.5) eFigure 3. Effect of the PTSD PRS on Educational Attainment (Yellow) and Cognitive Performance (Green) Considering Different Inclusion Thresholds eFigure 4. Leave-1-Out Analysis Conducted With Respect to the MathClass→PTSD2 Result eFigure 5. Identification of Potential Outliers (in Red) in MathClass Genetic Instrument Based on IVW Heterogeneity Test and MR-RAPS Standardized Residuals eFigure 6. Results of the MathClass→PTSD2 Analysis After the Removal of the Potential Outliers From the Genetic Instrument eFigure 7. Results of the Sensitivity Analyses With Respect to All MR Analyses Conducted eFigure 8. Multivariable Mendelian Randomization Analysis Considering the Effects [file jamanetwopen-2-e193447-s001.pdf]

Supplementary Online Content

Polimanti R, Ratanatharathorn A, Maihofer AX, et al; Psychiatric Genomics Consortium Posttraumatic Stress Disorder Working Group. Association of economic status and educational attainment with posttraumatic stress disorder: a mendelian randomization study. *JAMA Netw Open.* 2019;2(5):e193447.doi:10.1001/jamanetworkopen.2019.3447

eAppendix. Phenotype Definitions, Sample Overlap, Palindromic Variants, and Assortative Mating

eReferences.

eTable 1. Traumatic Experiences Assessed in the UK Biobank

eTable 2. Results of the Sensitivity Analyses Conducted With Respect to the MathClass→PTSD Test With and Without the Outlier Variants in the MathClass Genetic Instrument

eTable 3. Results of the IVW Analyses Considering Genetic Instruments With and Without Palindromic Variants With Ambiguous Allele Frequencies (PAL and noPAL, Respectively)

eTable 4. MR-RAPS Analysis Considering Various Adjustments Based on Genome-Wide Genetic Instruments

eTable 5. Results of the Sensitivity Analyses Conducted With Respect to the Income→PTSD and Risk-Tak→PTSD Tests

eTable 6. Genetic Correlation Among Trauma Experiences Assessed in the UK Biobank

eTable 7. Results (Causal Effects and Sensitivity Analyses) of the MR Test Conducted Using Trauma-Related Genetic Instruments With Respect to PTSD

eTable 8. Results of the Enrichment Analysis Based on Tissue-Specific and Cell Type–Specific Transcriptomic Data

eFigure 1. Schematic Workflow of the Analyses Conducted

eFigure 2. Genetic Correlations Estimated Between Traits Related to Cognitive Ability and 2 Versions of the Posttraumatic Stress Disorder Dataset, PGC-PTSD Freeze-2 (2) and PGC-PTSD Freeze-1.5 (1.5)

eFigure 3. Effect of the PTSD PRS on Educational Attainment (Yellow) and Cognitive Performance (Green) Considering Different Inclusion Thresholds

eFigure 4. Leave-1-Out Analysis Conducted With Respect to the MathClass→PTSD2 Result

© 2019 Polimanti R et al. *JAMA Network Open.*

eFigure 5. Identification of Potential Outliers (in Red) in MathClass Genetic Instrument Based on IVW Heterogeneity Test and MR-RAPS Standardized Residuals

eFigure 6. Results of the MathClass→PTSD2 Analysis After the Removal of the Potential Outliers From the Genetic Instrument

eFigure 7. Results of the Sensitivity Analyses With Respect to all MR Analyses Conducted

eFigure 8. Multivariable Mendelian Randomization Analysis Considering the Effects of EdAtt and MathClass on PTSD

eFigure 9. Effect of Income and RiskTak PRS (Green and Blue, Respectively) on PTSD Considering Different Inclusion Thresholds

eFigure 10. Genetic Correlations Between Traumatic Experiences and the Other Traits of Interest

eFigure 11. Effect of Trauma-Related PRS on PTSD Considering Different Inclusion Thresholds

eFigure 12. Multivariable Mendelian Randomization Analysis Considering the Effects of Physically Abused by Family as a Child and Belittlement by Partner or Ex-Partner as an Adult on PTSD

This supplementary material has been provided by the authors to give readers additional information about their work.

© 2019 Polimanti R et al. *JAMA Network Open.*

eAppendix. Phenotype Definitions, Sample Overlap, Palindromic Variants, and Assortative Mating

EdAtt – Phenotype Definition in the SSGAC study[1]

For each cohort included in the SSGAC GWAS meta-analysis,[1] each major educational qualification that can be identified from the cohort's survey measure was mapped to an International Standard Classification of Education (ISCED) category. To construct the EdAtt phenotype, SSGAC analysts imputed a years-of-education equivalent for each ISCED category. Across all SSGAC cohorts, the sample-size-weighted mean of EdAtt was 16.8 years of schooling with a standard deviation of 4.2.

Sample Overlap among the Cohorts Investigated

Some of the statistical methods used in the present analysis (i.e., polygenic risk scoring and MR) can be biased by sample overlap between the datasets investigated.[2,3] The main source of sample overlap between PGC-PTSD and SSGAC GWAS meta-analyses is the presence of some UK Biobank participants in both PGC-PTSD and SSGAC studies. For this reason, some analyses were conducted on a PGC-PTSD subsample excluding the UK Biobank cohort (PGC-PTSD freeze-1.5: 12,823 cases; 35,648 controls). Besides the UK Biobank, the only cohort included in both PGC-PTSD and SSGAC studies is QIMR (Queensland Institute of Medical Research). QIMR cohort represents 1.2% (325 cases and 1,797 controls) of the PGC-PTSD freeze-2 study and 0.7% (N = 8,006) of the SSGAC analysis. We believe that this minor overlap did not affect the results obtained. However, we verified the relationship of PTSD and traits related to educational attainment also considering that some traits were assessed only

© 2019 Polimanti R et al. *JAMA Network Open.*

in the 23andMe cohort, which is independent from PGC-PTSD and UK Biobank samples.

Palindromic Variants

Palindromic variants with an ambiguous allele frequency (i.e., minor allele frequency close to 50%) can introduce biases in the results generated by two-sample MR analyses.[4] We did not observe any difference in the causal effects observed using genetic instruments including or excluding these variants (Table S2).

Assortative Mating

Assortative mating has been documented in analyses of genome-wide SNP data on spousal pairs with respect to several traits, including educational attainment.[5] Since MR methods can be biased by this mechanism,[6] we confirmed the causal effect of EdAtt on PTSD (freeze-1.5; IVW beta = -0.32, $p = 9\times10^{-4}$) after correcting the analysis using the assortative-mating adjustment parameter calculated in the SSGAC GWAS.[1]

eReferences

1. Lee JJ, Wedow R, Okbay A, et al. Gene discovery and polygenic prediction from a genome-wide association study of educational attainment in 1.1 million individuals. *Nat Genet* 2018; **50**(8): 1112-21.
2. Choi SW, Mak TSH, Reilly P. A guide to performing Polygenic Risk Score analyses. *bioRxiv* 2018.
3. Burgess S, Davies NM, Thompson SG. Bias due to participant overlap in two-sample Mendelian randomization. *Genet Epidemiol* 2016; **40**(7): 597-608.
4. Hemani G, Tilling K, Davey Smith G. Orienting the causal relationship between imprecisely measured traits using GWAS summary data. *PLoS Genet* 2017; **13**(11): e1007081.
5. Robinson MR, Kleinman A, Graff M, et al. Genetic evidence of assortative mating in humans. *Nature Human Behaviour* 2017; **1**: 0016.

© 2019 Polimanti R et al. *JAMA Network Open.*

6. Hartwig FP, Davies NM, Davey Smith G. Bias in Mendelian randomization due to assortative mating. *Genet Epidemiol* 2018; **42**(7): 608-20.

© 2019 Polimanti R et al. *JAMA Network Open.*

eTable 1: Traumatic Experiences Assessed in the UK Biobank.

Field ID	Description	N (or case/controls)
20487	Felt hated by family member as a child	117,749
20488	Physically abused by family as a child	117,838
20489	Felt loved as a child	117,624
20490	Sexually molested as a child	116,773
20491	Someone to take to doctor when needed as a child	117,301
20521	Belittlement by partner or ex-partner as an adult	117,741
20522	Been in a confiding relationship as an adult	115,099
20523	Physical violence by partner or ex-partner as an adult	117,746
20524	Sexual interference by partner or ex-partner without consent as an adult	117,727
20525	Able to pay rent/mortgage as an adult	116,296
20526	Been in serious accident believed to be life-threatening	11,325/106597
20527	Been involved in combat or exposed to war-zone	4,010/113,944
20528	Diagnosed with life-threatening illness	19,291/98,326
20529	Victim of physically violent crime	21,926/95,920
20530	Witnessed sudden violent death	15,959/101,903
20531	Victim of sexual assault	17,230/99,441

© 2019 Polimanti R et al. *JAMA Network Open.*

eTable 2: Results of the Sensitivity Analyses Conducted With Respect to the MathClass→PTSD Test With and Without the Outlier Variants in the MathClass Genetic Instrument

**MR-Egger**	**Intercept**	**SE**	**P**
With-Outliers	0.003	0.007	0.668
Without-Outliers	0.003	0.006	0.608
**IVW Heterogeneity Test**	**Q**	**df**	**P**
With-Outliers	267.4	192	2.61E-04
Without-Outliers	160.2	174	0.765
**MR-RAPS**	**Estimated pleiotropy variance**	**SE**	**P**
With-Outliers	0.00011	4.11E-05	0.007
Without-Outliers	0	0	NaN
**MR-PRESSO Global test**	**RSSobs**	**P**	**Outliers N**
With-Outliers	281.4	5.00E-04	0
Without-Outliers	173.3	0.760	0

© 2019 Polimanti R et al. *JAMA Network Open.*

eTable 3: Results of the IVW Analyses Considering Genetic Instruments With and Without Palindromic Variants With Ambiguous Allele Frequencies (PAL and noPAL, Respectively)

Test	Genetic Instrument	Beta	SE	LCI	UCI
MathClass>PTSD2	PAL	-0.41	0.09	-0.58	-0.24
	noPAL	-0.40	0.09	-0.57	-0.23
MathClass>PTSD2_NO	PAL	-0.39	0.07	-0.53	-0.24
	noPAL	-0.37	0.08	-0.53	-0.22
MathClass>PTSD1.5	PAL	-0.25	0.09	-0.42	-0.08
	noPAL	-0.24	0.09	-0.41	-0.07
EdAtt1M>PTSD1.5	PAL	-0.22	0.08	-0.37	-0.07
	noPAL	-0.21	0.08	-0.37	-0.06
EdAtt>PTSD1.5	PAL	-0.26	0.08	-0.42	-0.11
	noPAL	-0.23	0.08	-0.39	-0.07

© 2019 Polimanti R et al. *JAMA Network Open.*

eTable 4: MR-RAPS Analysis Considering Various Adjustments Based on Genome-Wide Genetic Instruments (ie, All Informative LD-Independent Variants Are Included in the Genetic Instrument)

Over Dispersion	Loss Function	Beta	SE	P
EdAtt→PTSD				
FALSE	l2	-0.27	0.06	1.80E-06
FALSE	huber	-0.27	0.06	2.61E-06
FALSE	tukey	-0.27	0.06	2.79E-06
TRUE	l2	-0.26	0.06	6.39E-06
TRUE	huber	-0.27	0.06	2.61E-06
TRUE	tukey	-0.27	0.06	2.79E-06
PTSD→EdAtt				
FALSE	l2	-0.0013	0.0008	0.117
FALSE	huber	-0.0011	0.0008	0.196
FALSE	tukey	-0.001	0.0008	0.224
TRUE	l2	-0.0003	0.001	0.763
TRUE	huber	-0.0005	0.001	0.660
TRUE	tukey	-0.0005	0.001	0.648

© 2019 Polimanti R et al. *JAMA Network Open.*

eTable 5: Results of the Sensitivity Analyses Conducted With Respect to the Income→PTSD and Risk-Tak→PTSD Tests

**MR-Egger**	**Intercept**	**SE**	**P**
Income→PTSD	-0.003	0.003	0.333
Risk-Tak→PTSD	0.005	0.004	0.247
**IVW Heterogeneity Test**	**Q**	**df**	**P**
Income→PTSD	660.4	632	0.210
Risk-Tak→PTSD	234.3	277	0.970
**MR-RAPS**	**Estimated pleiotropy variance**	**SE**	**P**
Income→PTSD	3.32E-05	2.76E-05	0.333
Risk-Tak→PTSD	0	0	NaN
**MR-PRESSO Global test**	**RSSobs**	**P**	**Outliers N**
Income→PTSD	680.7	0.166	0
Risk-Tak→PTSD	240.6	0.978	0

© 2019 Polimanti R et al. *JAMA Network Open.*

eTable 6: Genetic Correlation Among Trauma Experiences Assessed in the UK Biobank

Phenotype1-Phenotype2	r_g	SE	P	FDR Q
20487-20489	-0.8399	0.0316	1.59E-155	1.91E-153
20487-20488	0.9207	0.0387	2.43E-125	1.46E-123
20488-20489	-0.7443	0.0355	1.24E-97	4.96E-96
20521-20523	0.8238	0.0491	4.58E-63	1.37E-61
20487-20521	0.8924	0.0557	1.14E-57	2.74E-56
20490-20531	0.9294	0.0645	4.22E-47	8.44E-46
20489-20521	-0.7114	0.0497	1.91E-46	3.27E-45
20488-20521	0.7503	0.0589	3.38E-37	5.07E-36
20489-20491	0.6571	0.0534	8.92E-35	1.19E-33
20488-20531	0.7714	0.064	1.94E-33	2.33E-32
20523-20524	0.9313	0.0803	4.50E-31	4.91E-30
20489-20523	-0.733	0.0647	9.08E-30	9.08E-29
20487-20523	0.8611	0.0766	2.36E-29	2.18E-28
20488-20523	0.8143	0.0749	1.49E-27	1.28E-26
20521-20531	0.7104	0.0681	1.71E-25	1.37E-24
20521-20524	0.9425	0.0921	1.33E-24	9.98E-24
20487-20531	0.6732	0.0686	1.01E-22	7.13E-22
20488-20490	0.7403	0.0766	4.38E-22	2.92E-21
20489-20531	-0.572	0.0597	9.49E-22	5.99E-21
20487-20490	0.7335	0.0852	7.60E-18	4.56E-17
20491-20525	0.7668	0.0915	5.29E-17	3.02E-16
20529-20531	0.8703	0.1047	9.39E-17	5.12E-16
20489-20490	-0.541	0.0687	3.35E-15	1.75E-14
20523-20531	0.7001	0.0915	1.95E-14	9.75E-14
20490-20521	0.646	0.0855	4.25E-14	2.04E-13
20524-20531	0.9057	0.1206	6.05E-14	2.79E-13
20488-20529	0.5572	0.075	1.11E-13	4.93E-13
20487-20491	-0.5185	0.0707	2.24E-13	9.60E-13
20491-20523	-0.6919	0.0954	4.13E-13	1.71E-12
20491-20521	-0.5721	0.0805	1.16E-12	4.64E-12
20490-20523	0.7469	0.1066	2.42E-12	9.37E-12
20489-20522	0.4133	0.0595	3.71E-12	1.39E-11
20488-20491	-0.4737	0.0704	1.74E-11	6.33E-11
20489-20524	-0.6631	0.1015	6.33E-11	2.23E-10
20526-20529	1	0.1706	9.55E-11	3.27E-10
20488-20530	0.5655	0.0878	1.16E-10	3.87E-10

© 2019 Polimanti R et al. *JAMA Network Open.*

20526-20531	0.8114	0.1282	2.49E-10	8.08E-10
20523-20525	-0.6959	0.1108	3.42E-10	1.08E-09
20530-20531	0.614	0.099	5.50E-10	1.69E-09
20523-20529	0.6458	0.1068	1.46E-09	4.38E-09
20488-20527	0.7115	0.118	1.64E-09	4.80E-09
20490-20524	0.8124	0.1356	2.11E-09	6.03E-09
20487-20524	0.6334	0.1062	2.47E-09	6.89E-09
20487-20530	0.5684	0.0959	3.08E-09	8.40E-09
20526-20530	0.8198	0.1411	6.26E-09	1.67E-08
20521-20529	0.5158	0.0896	8.73E-09	2.28E-08
20489-20526	-0.5582	0.0978	1.13E-08	2.89E-08
20489-20529	-0.4056	0.0711	1.17E-08	2.93E-08
20488-20524	0.5921	0.105	1.72E-08	4.21E-08
20529-20530	0.6178	0.1099	1.92E-08	4.61E-08
20521-20526	0.6381	0.1158	3.58E-08	8.42E-08
20487-20529	0.4865	0.0887	4.10E-08	9.46E-08
20491-20522	0.4983	0.091	4.32E-08	9.78E-08
20487-20526	0.6625	0.1262	1.52E-07	3.38E-07
20489-20530	-0.3474	0.0664	1.67E-07	3.64E-07
20488-20526	0.5618	0.111	4.13E-07	8.85E-07
20524-20529	0.6632	0.1361	1.10E-06	2.32E-06
20524-20526	0.8346	0.1762	2.18E-06	4.51E-06
20487-20522	-0.3456	0.0736	2.62E-06	5.33E-06
20489-20525	0.3436	0.0736	3.06E-06	6.12E-06
20490-20530	0.5835	0.1262	3.76E-06	7.40E-06
20527-20530	0.6581	0.1428	4.06E-06	7.86E-06
20488-20525	-0.3998	0.0884	6.15E-06	1.17E-05
20490-20526	0.6938	0.154	6.67E-06	1.25E-05
20521-20530	0.4157	0.0926	7.21E-06	1.33E-05
20487-20527	0.5476	0.1228	8.26E-06	1.50E-05
20523-20526	0.6348	0.1436	9.90E-06	1.77E-05
20523-20530	0.4685	0.1087	1.65E-05	2.91E-05
20526-20527	0.9542	0.2243	2.09E-05	3.63E-05
20527-20531	0.5404	0.1281	2.47E-05	4.23E-05
20491-20531	-0.3507	0.0842	3.11E-05	5.26E-05
20524-20530	0.5885	0.1417	3.28E-05	5.46E-05
20526-20528	0.853	0.2055	3.32E-05	5.46E-05
20523-20527	0.5835	0.1497	9.75E-05	0.000156
20521-20525	-0.3805	0.1001	0.0001	0.000156
20487-20525	-0.3492	0.0917	0.0001	0.000156
20490-20529	0.4375	0.1129	0.0001	0.000156

© 2019 Polimanti R et al. *JAMA Network Open.*

20490-20491	-0.4118	0.1093	0.0002	0.000296
20521-20528	0.4716	0.1284	0.0002	0.000296
20489-20527	-0.3946	0.1048	0.0002	0.000296
20491-20530	-0.417	0.112	0.0002	0.000296
20522-20523	-0.3333	0.0915	0.0003	0.000429
20524-20525	-0.5356	0.147	0.0003	0.000429
20521-20527	0.4853	0.1334	0.0003	0.000429
20527-20529	0.5639	0.1624	0.0005	0.000682
20528-20531	0.4717	0.1352	0.0005	0.000682
20488-20522	-0.2382	0.0682	0.0005	0.000682
20488-20528	0.43	0.1237	0.0005	0.000682
20522-20525	0.3549	0.1029	0.0006	8.00E-04
20491-20527	-0.4869	0.1415	0.0006	8.00E-04
20487-20528	0.3784	0.1114	0.0007	0.000923
20523-20528	0.4946	0.1521	0.0011	0.001419
20490-20528	0.4906	0.1498	0.0011	0.001419
20490-20527	0.5086	0.1598	0.0015	0.001915
20528-20530	0.497	0.1585	0.0017	0.002147
20489-20528	-0.3004	0.0965	0.0019	0.002375
20528-20529	0.4998	0.1642	0.0023	0.002845
20524-20527	0.5686	0.1883	0.0025	0.003061
20525-20530	-0.3357	0.117	0.0041	0.00497
20491-20524	-0.3506	0.1244	0.0048	0.00576
20491-20526	-0.3962	0.1423	0.0054	0.006416
20491-20528	-0.3578	0.1355	0.0083	0.009765
20524-20528	0.487	0.1998	0.0148	0.017243
20521-20522	-0.1879	0.0808	0.0201	0.023192
20525-20531	-0.2388	0.1037	0.0213	0.024343
20490-20525	-0.2303	0.117	0.0491	0.055585
20525-20527	-0.2874	0.1555	0.0646	0.072449
20527-20528	0.307	0.179	0.0862	0.095778
20491-20529	-0.1828	0.1107	0.0987	0.108661
20522-20524	-0.1516	0.1135	0.1816	0.198109
20525-20529	0.1311	0.1135	0.2482	0.268324
20522-20531	-0.094	0.0864	0.2766	0.296357
20525-20528	-0.1683	0.1609	0.2958	0.314124
20522-20529	0.1056	0.1025	0.3028	0.318737
20525-20526	-0.1249	0.1605	0.4366	0.455583
20490-20522	-0.059	0.1023	0.5642	0.583655
20522-20527	-0.0608	0.1211	0.6154	0.63118
20522-20530	0.0292	0.0863	0.7352	0.747661

© 2019 Polimanti R et al. *JAMA Network Open.*

20522-20528	-0.0026	0.138	0.9848	0.9928
20522-20526	0.0011	0.1264	0.9928	0.9928

© 2019 Polimanti R et al. *JAMA Network Open.*

eTable 7: Results (Causal Effects and Sensitivity Analyses) of the MR Test Conducted Using Trauma-Related Genetic Instruments With Respect to PTSD

**Exposure** **Method**		**Felt hated by family member as a child**	**Physically abused by family as a child**	**Felt loved as a child**	**Belittlement by partner or ex-partner as an adult**
**IVW**	Beta	0.212	0.356	-0.255	0.172
	SE	0.072	0.085	0.052	0.059
	PI	0.003	2.57E-05	7.06E-07	0.003
**IVW Heterogeneity Test**	Q	449.0	453.4	461.0	415.2
	df	425	410	458	445
	P	0.203	0.068	0.452	0.841
**MR-Egger**	BETA	0.493	0.033	0.110	0.057
	SE	0.151	0.191	0.122	0.124
	PI	0.001	0.863	0.366	0.643
**MR-Egger Intercept**	Intercept	-0.007	0.007	-0.010	0.003
	SE	0.003	0.003	0.003	0.003
	P	0.035	0.060	0.001	0.291
**MR-RAPS**	Beta	0.238	0.373	-0.281	0.173
	SE	0.080	0.093	0.057	0.062
	P	0.003	6.07E-05	8.78E-07	0.005
**MR-RAPS Pleiotropy Test**	Variance	6.65E-05	1.03E-04	2.45E-05	0
	SE	5.62E-05	5.65E-05	4.05E-05	0
	P	0.236	0.069	0.546	NaN
**MR-PRESSO**	Beta	0.205	0.355	-0.260	0.141
	SE	0.072	0.084	0.051	0.054
	P	0.005	2.99E-05	5.35E-07	0.010
**MR-PRESSO Global Test**	RSSobs	452.6324	462.4988	466.4951	428.5089
	P	0.225	0.066	0.470	0.778

© 2019 Polimanti R et al. *JAMA Network Open.*

eTable 8: Results of the Enrichment Analysis Based on Tissue-Specific and Cell Type–Specific Transcriptomic Data

Trait	EdAtt		Income	
GTEx				
**Tissue**	**BETA**	**P**	**BETA**	**P**
Brain_Cerebellar_Hemisphere	0.0982	1.49E-16	0.0395	3.83E-07
Brain_Cerebellum	0.1	1.21E-15	0.0403	6.76E-07
Brain_Frontal_Cortex_BA9	0.0983	6.62E-13	0.0414	2.78E-06
Brain_Cortex	0.0975	9.20E-12	0.0411	7.88E-06
Brain_Anterior_cingulate_cortex_BA24	0.0919	3.07E-10	0.0397	2.47E-05
Brain_Nucleus_accumbens_basal_ganglia	0.0864	1.27E-08	0.0367	0.000145
Brain_Hippocampus	0.0874	6.24E-08	0.0381	0.000197
Brain_Amygdala	0.0806	1.96E-07	0.0347	0.000429
Brain_Hypothalamus	0.0859	3.80E-07	0.0366	0.000434
Brain_Caudate_basal_ganglia	0.077	1.71E-06	0.0327	0.001168
Brain_Putamen_basal_ganglia	0.0717	3.81E-06	0.0297	0.002422
Pituitary	0.0694	0.000776	0.0298	0.011664
Brain_Substantia_nigra	0.0579	0.000987	0.0248	0.0183
Brain_Spinal_cord_cervical_c-1	0.0485	0.007807	0.027	0.015214
Testis	0.0251	0.013298	0.0159	0.01622
Cells_EBV-transformed_lymphocytes	0.00632	0.23595	0.00765	0.10632
Cells_Transformed_fibroblasts	0.000486	0.48597	0.00913	0.15612
Adrenal_Gland	-0.00383	0.56375	-0.0173	0.88267
Ovary	-0.00584	0.59539	0.015	0.15093
Muscle_Skeletal	-0.00493	0.64895	-0.00755	0.80473

© 2019 Polimanti R et al. *JAMA Network Open.*

Uterus	-0.0313	0.8422	0.00294	0.43525
Colon_Sigmoid	-0.0381	0.84845	-0.00345	0.56596
Esophagus_Gastroesophageal_Junction	-0.0475	0.88722	-0.0121	0.70874
Esophagus_Muscularis	-0.0451	0.88997	-0.0127	0.72949
Nerve_Tibial	-0.0358	0.89334	-0.0173	0.85216
Heart_Atrial_Appendage	-0.0317	0.93275	-0.0399	0.99853
Artery_Tibial	-0.043	0.94571	-0.00671	0.66491
Heart_Left_Ventricle	-0.0305	0.95833	-0.0378	0.99931
Pancreas	-0.03	0.96553	-0.0162	0.92949
Cervix_Ectocervix	-0.0742	0.96915	-0.0122	0.70887
Whole_Blood	-0.0205	0.98382	-0.0131	0.97082
Thyroid	-0.055	0.98426	-0.00688	0.67599
Cervix_Endocervix	-0.0765	0.98785	-0.0154	0.78747
Fallopian_Tube	-0.081	0.98912	-0.0217	0.86041
Artery_Aorta	-0.0647	0.99042	-0.0149	0.82158
Artery_Coronary	-0.0861	0.99241	-0.035	0.96024
Liver	-0.0298	0.99505	-0.0247	0.99895
Bladder	-0.0888	0.9951	-0.0452	0.98972
Vagina	-0.083	0.99564	-0.0341	0.96842
Prostate	-0.0991	0.99698	-0.0385	0.97004
Skin_Not_Sun_Exposed_Suprapubic	-0.0501	0.99786	-0.0232	0.98138
Skin_Sun_Exposed_Lower_leg	-0.0516	0.99844	-0.0232	0.98177
Stomach	-0.0949	0.99867	-0.0441	0.99077
Adipose_Subcutaneous	-0.0926	0.99913	-0.0454	0.99592
Esophagus_Mucosa	-0.0532	0.99939	-0.0296	0.99724
Spleen	-0.0513	0.99943	-0.0207	0.97981
Breast_Mammary_Tissue	-0.132	0.99964	-0.0696	0.99924
Colon_Transverse	-0.103	0.99968	-0.0397	0.9879
Kidney_Cortex	-0.074	0.99981	-0.044	0.99962
Small_Intestine_Terminal_Ileum	-0.0804	0.99985	-0.0287	0.98337
Adipose_Visceral_Omentum	-0.118	0.99993	-0.0586	0.99941
Minor_Salivary_Gland	-0.0999	0.99998	-0.0513	0.99976
Lung	-0.104	1	-0.0448	0.99926
**Cortex**				
**Cell type**	**BETA**	**P**	**BETA**	**P**
neurons	0.0804	1.70E-06	0.0399	0.00018
fetal_quiescent	0.06	0.000111	0.0192	0.044132
hybrid	0.0938	0.00024	0.0378	0.009398

© 2019 Polimanti R et al. *JAMA Network Open.*

oligodendrocytes	-0.00061	0.51667	0.00566	0.287
OPC	-0.0114	0.75919	-0.0158	0.92398
fetal_replicating	-0.0138	0.81459	-0.00229	0.58443
astrocytes	-0.0161	0.8728	-0.00731	0.77638
microglia	-0.0474	0.99994	-0.0235	0.99536
endothelial	-0.0753	1	-0.0269	0.9962
**Hippocampus**				
**Cell type**	**BETA**	**P**	**BETA**	**P**
GABA2	1.47	4.23E-06	0.894	4.24E-05
exCA1	1.33	1.07E-05	0.464	0.014588
exPFC1	1.41	9.71E-05	0.595	0.010831
GABA1	1.24	0.000285	0.718	0.001726
exDG	0.941	0.000542	0.498	0.007281
exPFC2	0.836	0.00143	0.301	0.052282
exCA3	0.618	0.019504	0.0964	0.32455
OPC	-0.333	0.78642	-0.0024	0.50313
ODC1	-0.41	0.9072	-0.133	0.73382
ODC2	-0.496	0.9702	-0.0715	0.64686
ASC1	-0.907	0.99836	-0.563	0.9925
MG	-1.03	0.99839	-0.625	0.98971
NSC	-0.393	0.99867	-0.227	0.98834
ASC2	-0.896	0.99968	-0.681	0.99952
END	-0.642	1	-0.182	0.96368
**Pre-frontal Cortex**				
**Cell type**	**BETA**	**P**	**BETA**	**P**
GABAergic_neurons	0.14	8.96E-05	0.0488	0.017151
Astrocytes	0.0161	0.2512	-0.00241	0.56252
Neurons	0.00216	0.47808	0.00402	0.43326
OPC	-0.0116	0.60234	-0.00836	0.62414
Stem_cells	-0.0404	0.93612	0.00623	0.3562
Microglia	-0.0275	0.95191	-0.0147	0.9053
**Midbrain**				
**Cell type**	**BETA**	**P**	**BETA**	**P**
Gaba	0.713	5.23E-17	0.3	5.76E-07

© 2019 Polimanti R et al. *JAMA Network Open.*

NbGaba	0.595	3.56E-16	0.264	3.46E-07
DA1	0.481	1.08E-09	0.141	0.006199
NbML5	0.679	1.24E-08	0.344	1.89E-05
Sert	0.198	0.00011	0.0729	0.02764
DA0	0.376	0.001459	0.0801	0.17967
RN	0.311	0.002557	0.156	0.021646
DA2	0.208	0.011987	0.0173	0.39402
NbM	0.277	0.026398	0.136	0.077594
NbML1	0.233	0.042149	-0.00043	0.50192
OMTN	0.042	0.36092	-0.101	0.90238
Rgl2b	-0.016	0.55399	0.0352	0.33043
Rgl1	-0.0731	0.7576	0.0589	0.20091
OPC	-0.083	0.8201	-0.0327	0.69907
Rgl2c	-0.12	0.91998	-0.0498	0.80025
Rgl2a	-0.163	0.96156	-0.0267	0.66654
Rgl3	-0.142	0.9744	-0.0617	0.89154
NProg	-0.366	0.99535	-0.122	0.9029
ProgFPL	-0.227	0.99689	-0.0527	0.82512
Mgl	-0.168	0.99854	-0.0849	0.9805
ProgBP	-0.472	0.99997	-0.146	0.96734
ProgM	-0.474	0.99999	-0.131	0.96222
Endo	-0.28	1	-0.0964	0.99527
Peric	-0.3	1	-0.116	0.99723
ProgFPM	-0.429	1	-0.226	0.99982

© 2019 Polimanti R et al. *JAMA Network Open.*

eFigure 1: Schematic Workflow of the Analyses Conducted

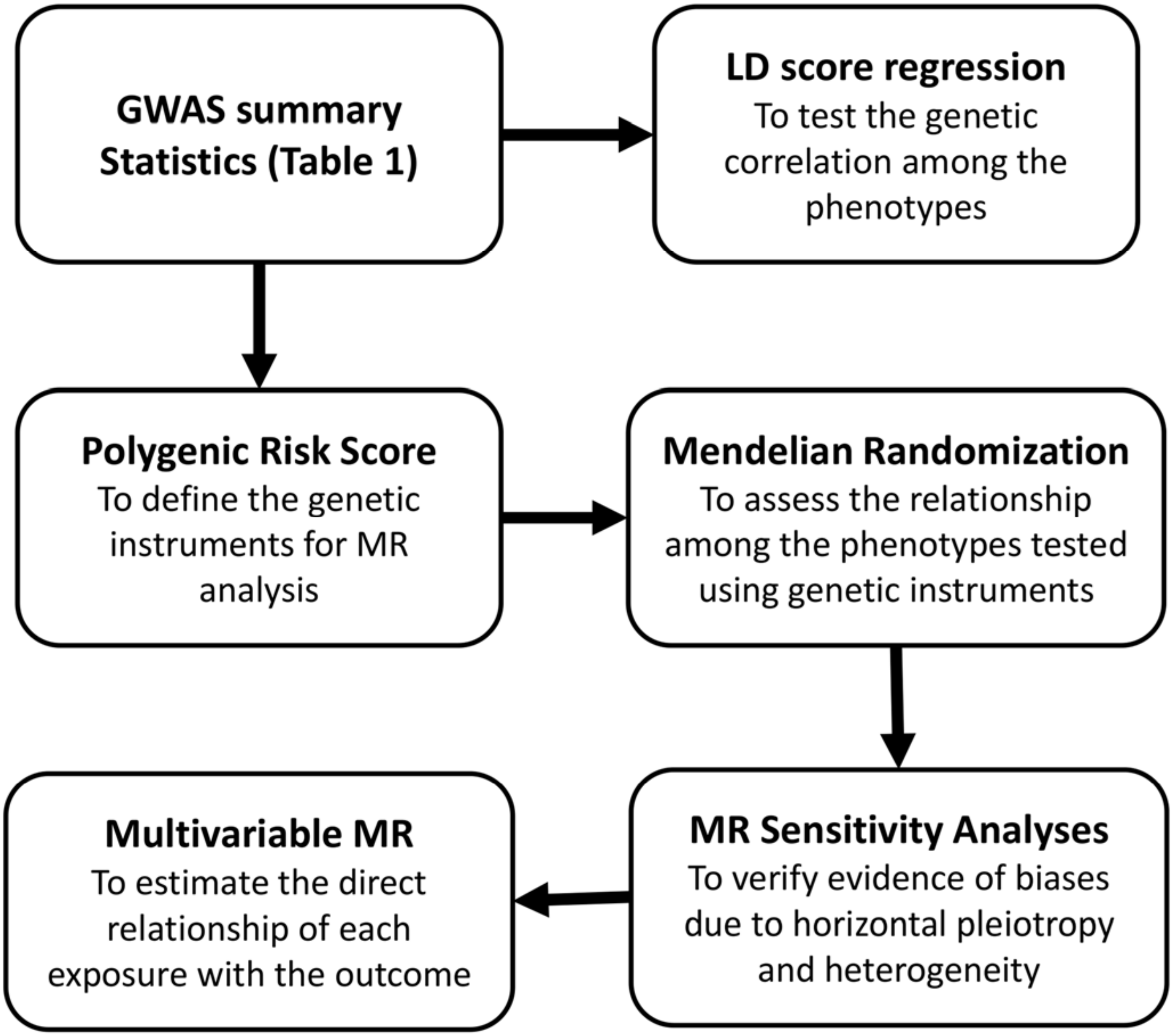

© 2019 Polimanti R et al. *JAMA Network Open.*

eFigure 2: Genetic Correlations Estimated Between Traits Related to Cognitive Ability and 2 Versions of the Posttraumatic Stress Disorder Dataset, PGC-PTSD Freeze-2 (2) and PGC-PTSD Freeze-1.5 (1.5)

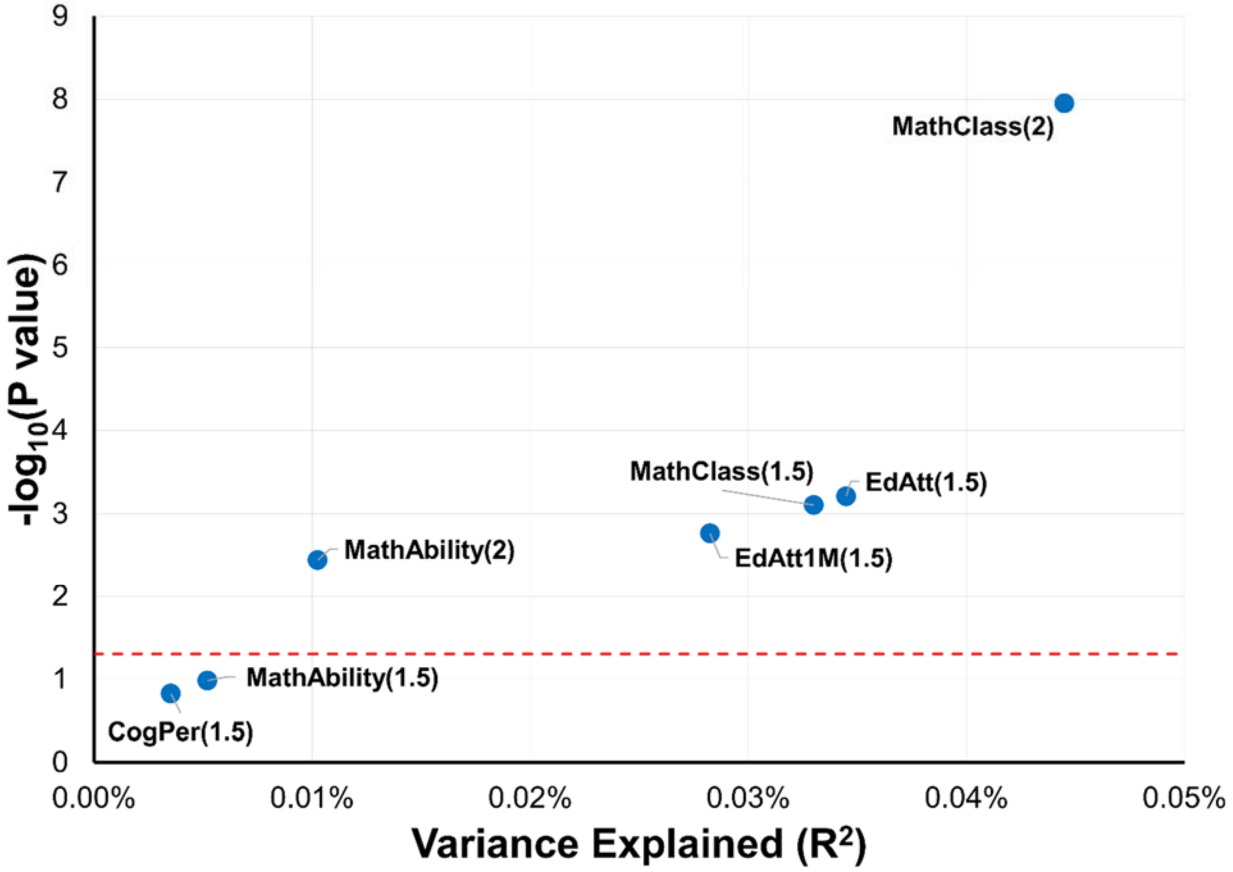

Red dotted line corresponds to nominal significance ($p<0.05$)

© 2019 Polimanti R et al. *JAMA Network Open.*

eFigure 3: Effect of the PTSD PRS on Educational Attainment (Yellow) and Cognitive Performance (Green) Considering Different Inclusion Thresholds

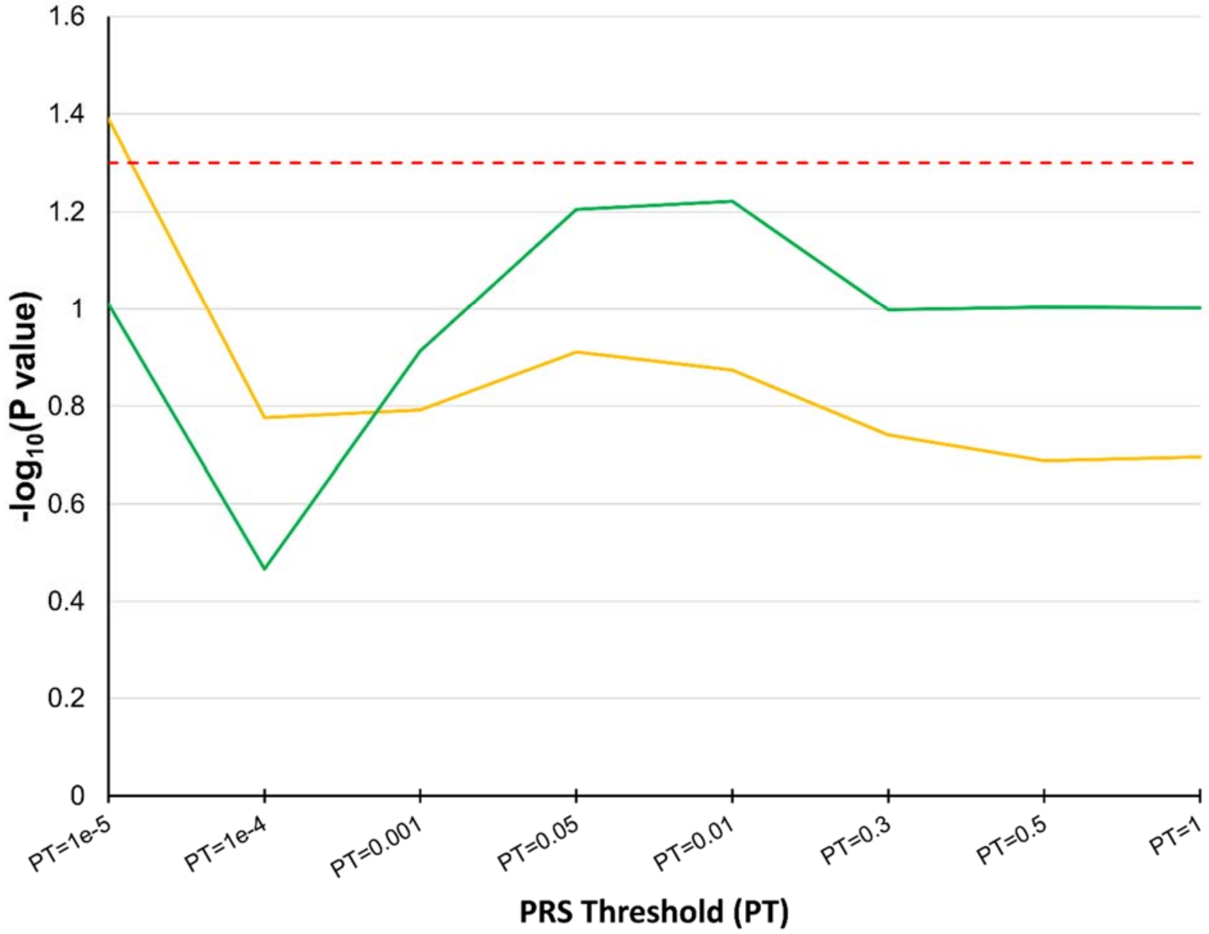

Red dotted line corresponds to nominal significance ($p<0.05$).

© 2019 Polimanti R et al. *JAMA Network Open.*

eFigure 4: Leave-1-Out Analysis Conducted With Respect to the MathClass→PTSD2 Result

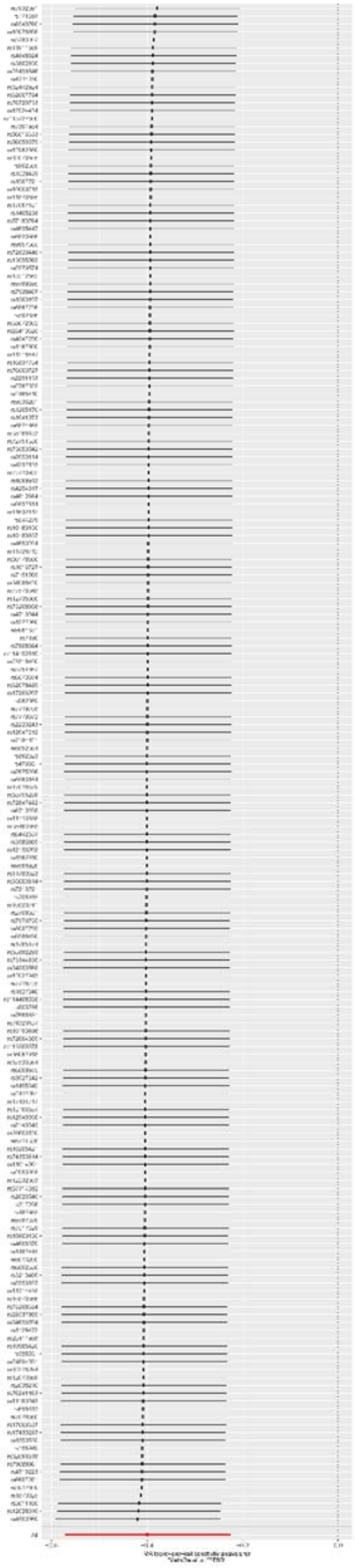

© 2019 Polimanti R et al. *JAMA Network Open.*

eFigure 5: Identification of Potential Outliers (in Red) in MathClass Genetic Instrument Based on IVW Heterogeneity Test and MR-RAPS Standardized Residuals

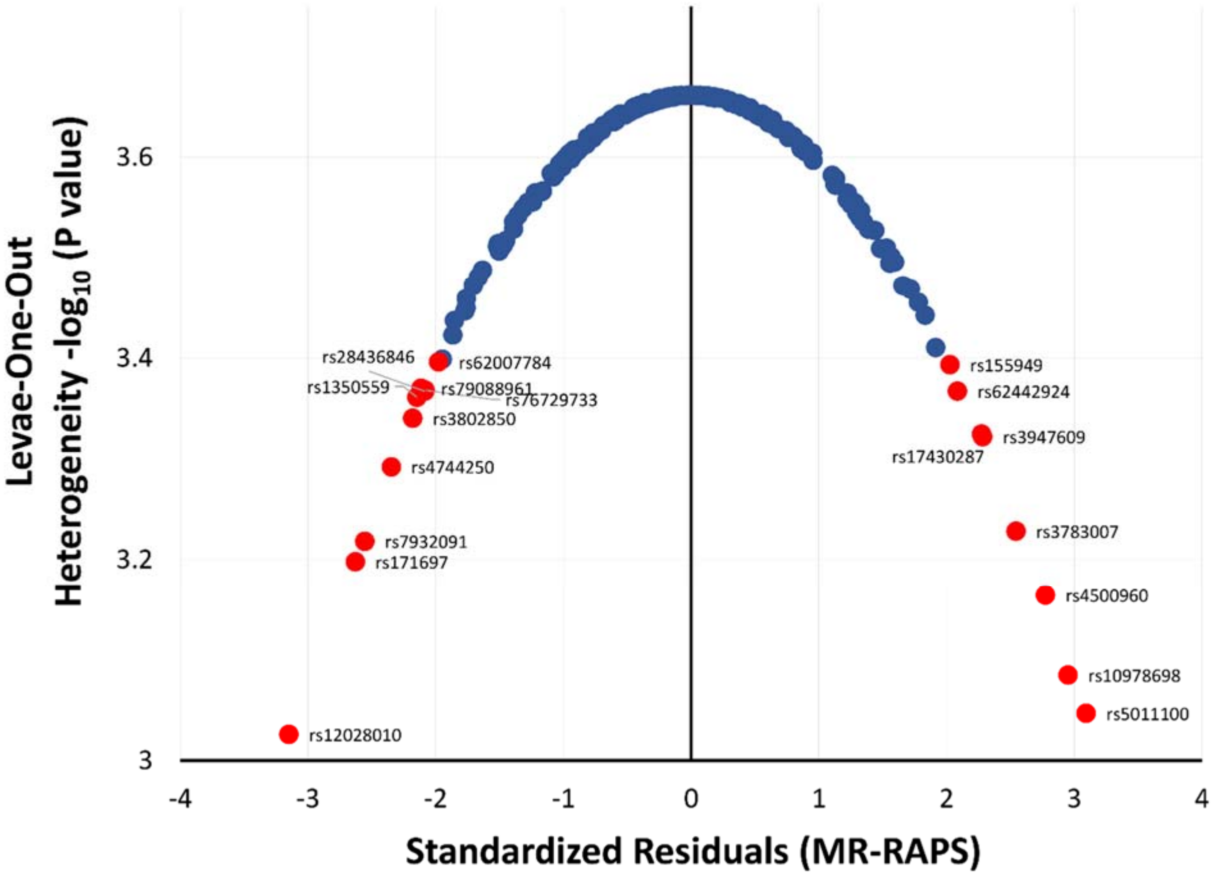

© 2019 Polimanti R et al. *JAMA Network Open.*

eFigure 6: Results of the MathClass→PTSD2 Analysis After the Removal of the Potential Outliers From the Genetic Instrument

A

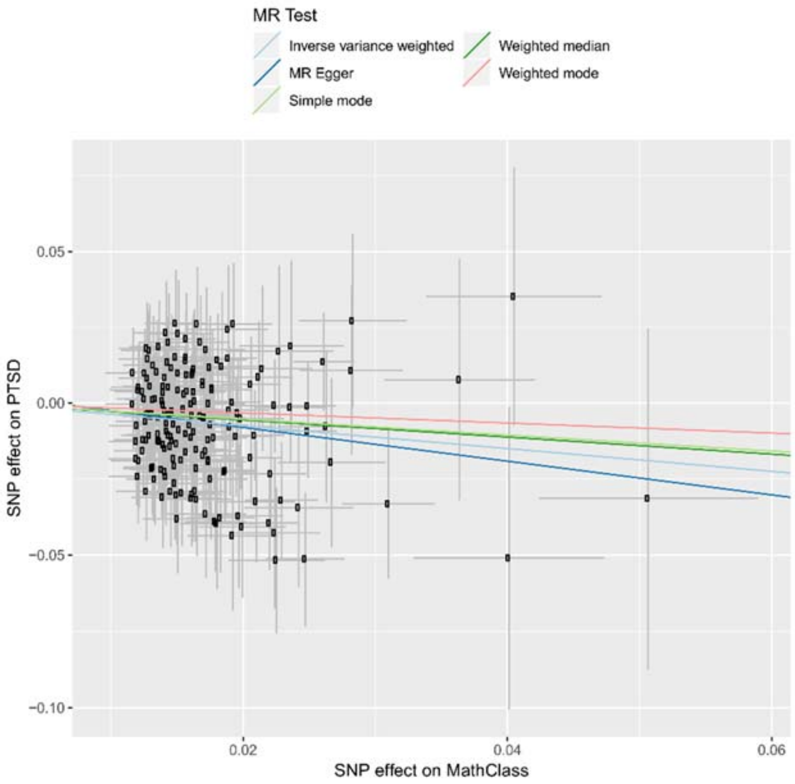

B

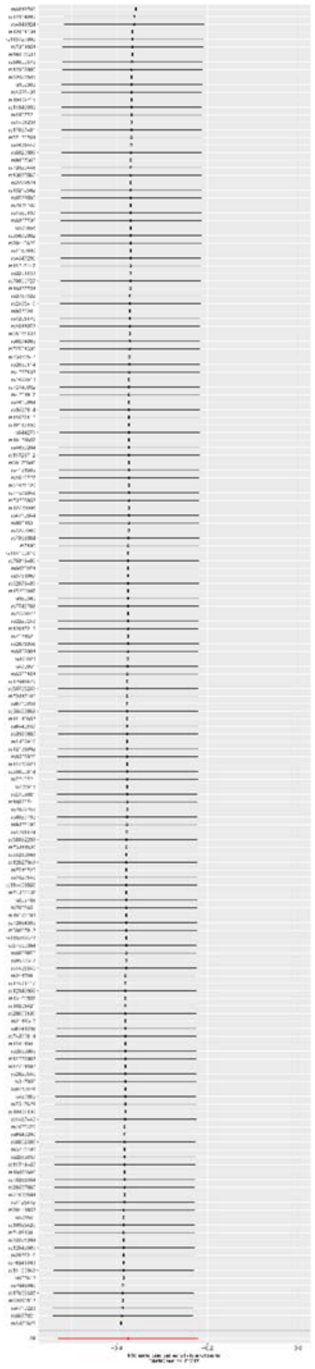

A. SNP-exposure (MathClass associations, beta) and SNP-outcome (PTSD freeze-2 associations, logOR) coefficients used in the MR. Error bars (95% CIs) are reported for each association. **B**. Leave-one-out analysis conducted with respect to the result excluding the potential outliers from the genetic instrument.

© 2019 Polimanti R et al. *JAMA Network Open.*

eFigure 7: Results of the Sensitivity Analyses With Respect to all MR Analyses Conducted

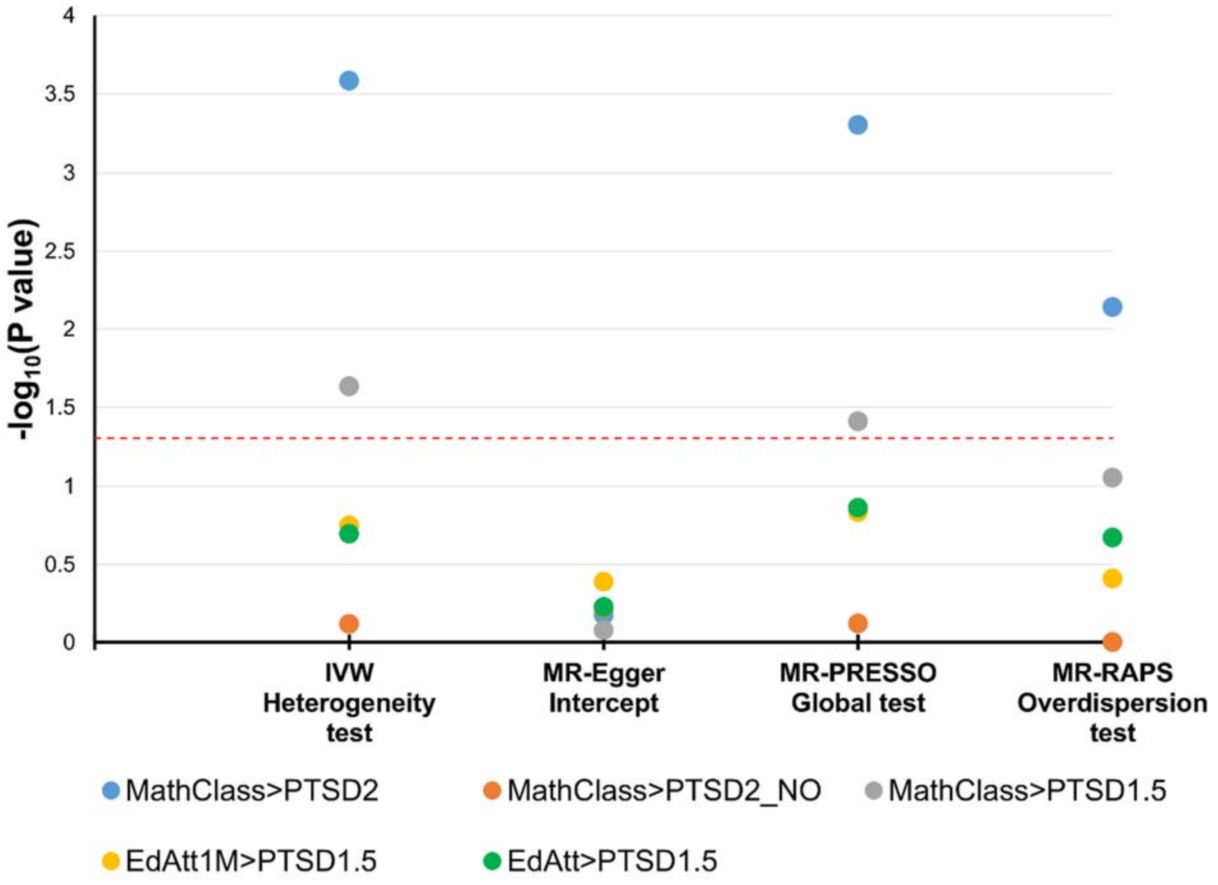

Red dotted line corresponds to nominal significance ($p<0.05$).

© 2019 Polimanti R et al. *JAMA Network Open.*

eFigure 8: Multivariable Mendelian Randomization Analysis Considering the Effects of EdAtt and MathClass on PTSD

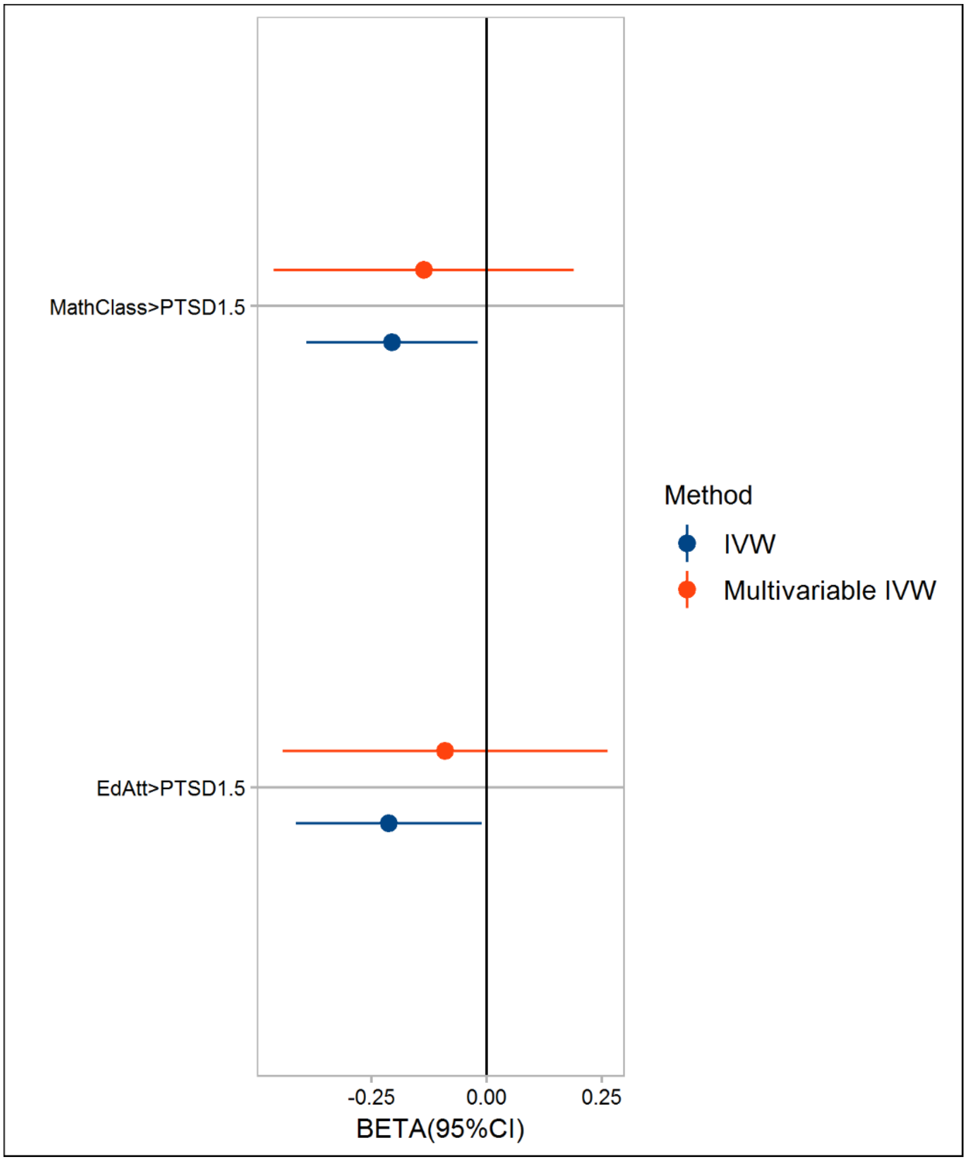

© 2019 Polimanti R et al. *JAMA Network Open.*

eFigure 9: Effect of Income and RiskTak PRS (Green and Blue, Respectively) on PTSD Considering Different Inclusion Thresholds

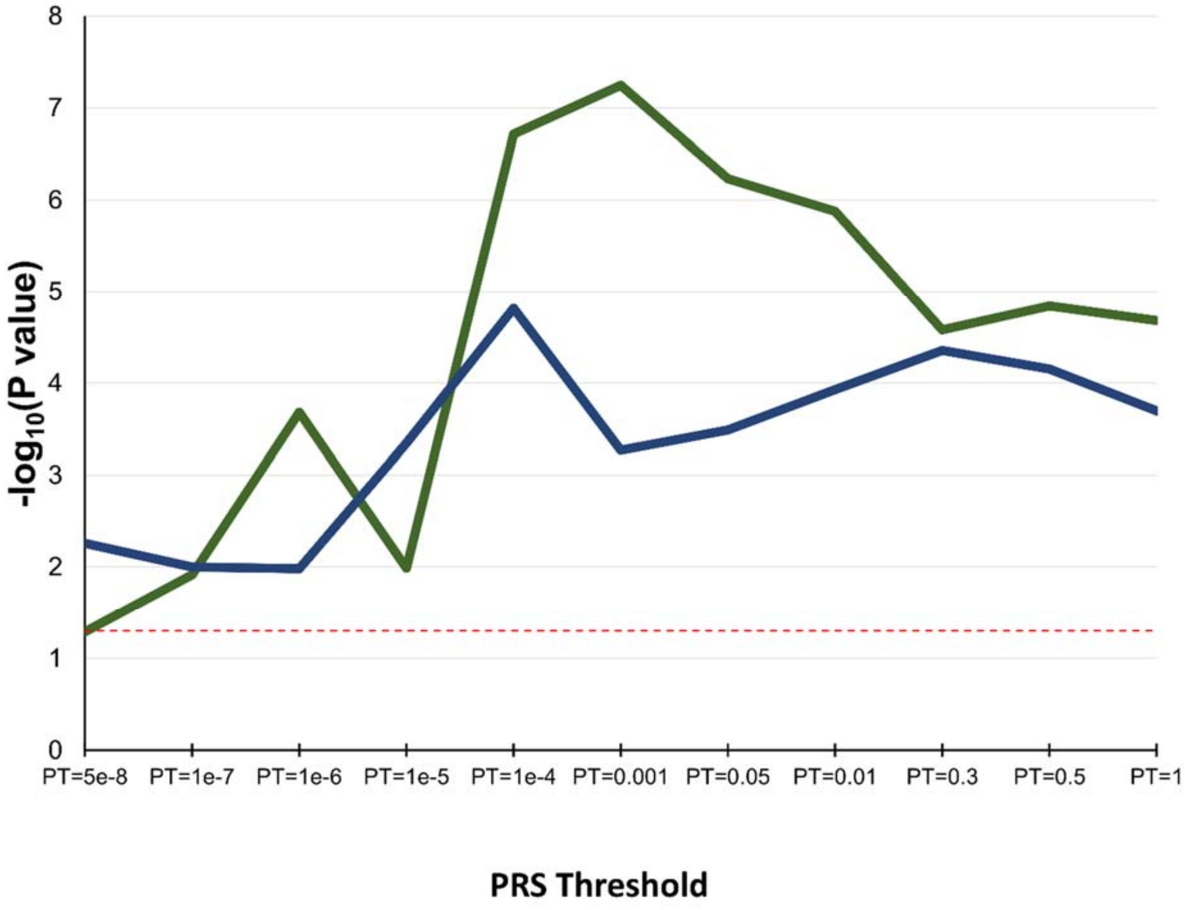

Red dotted line corresponds to nominal significance ($p<0.05$).

© 2019 Polimanti R et al. *JAMA Network Open.*

eFigure 10: Genetic Correlations Between Traumatic Experiences and the Other Traits of Interest

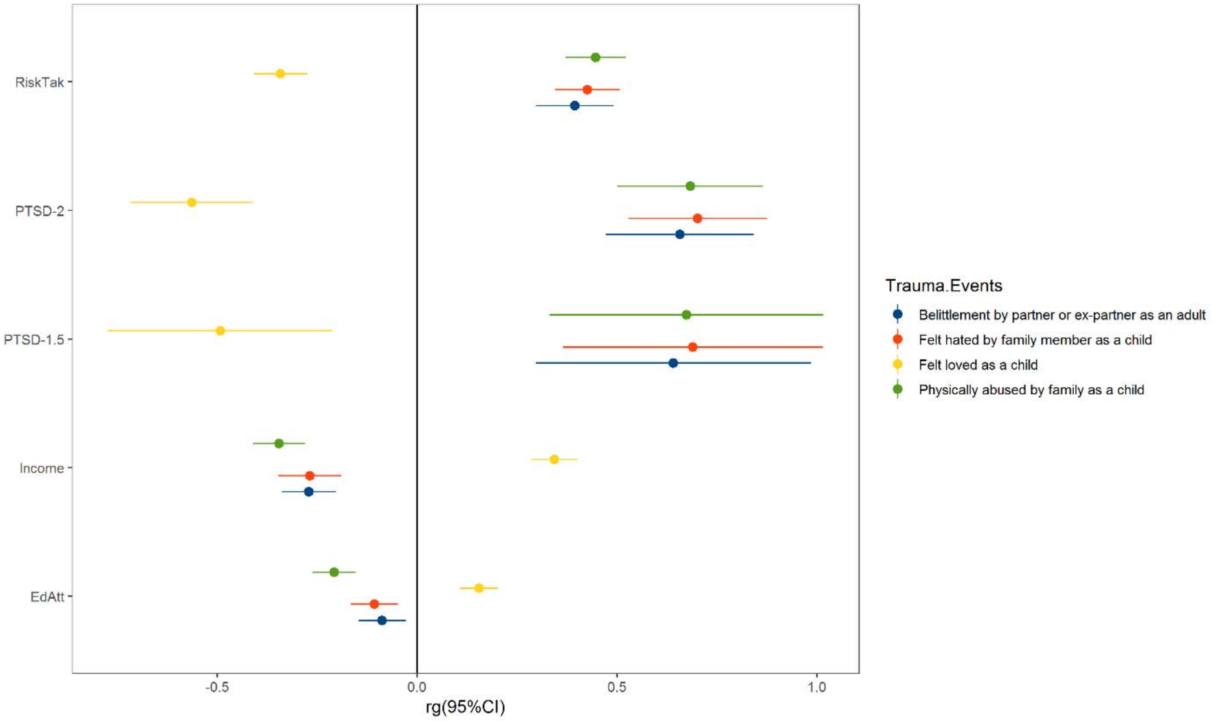

© 2019 Polimanti R et al. *JAMA Network Open.*

eFigure 11: Effect of Trauma-Related PRS on PTSD Considering Different Inclusion Thresholds

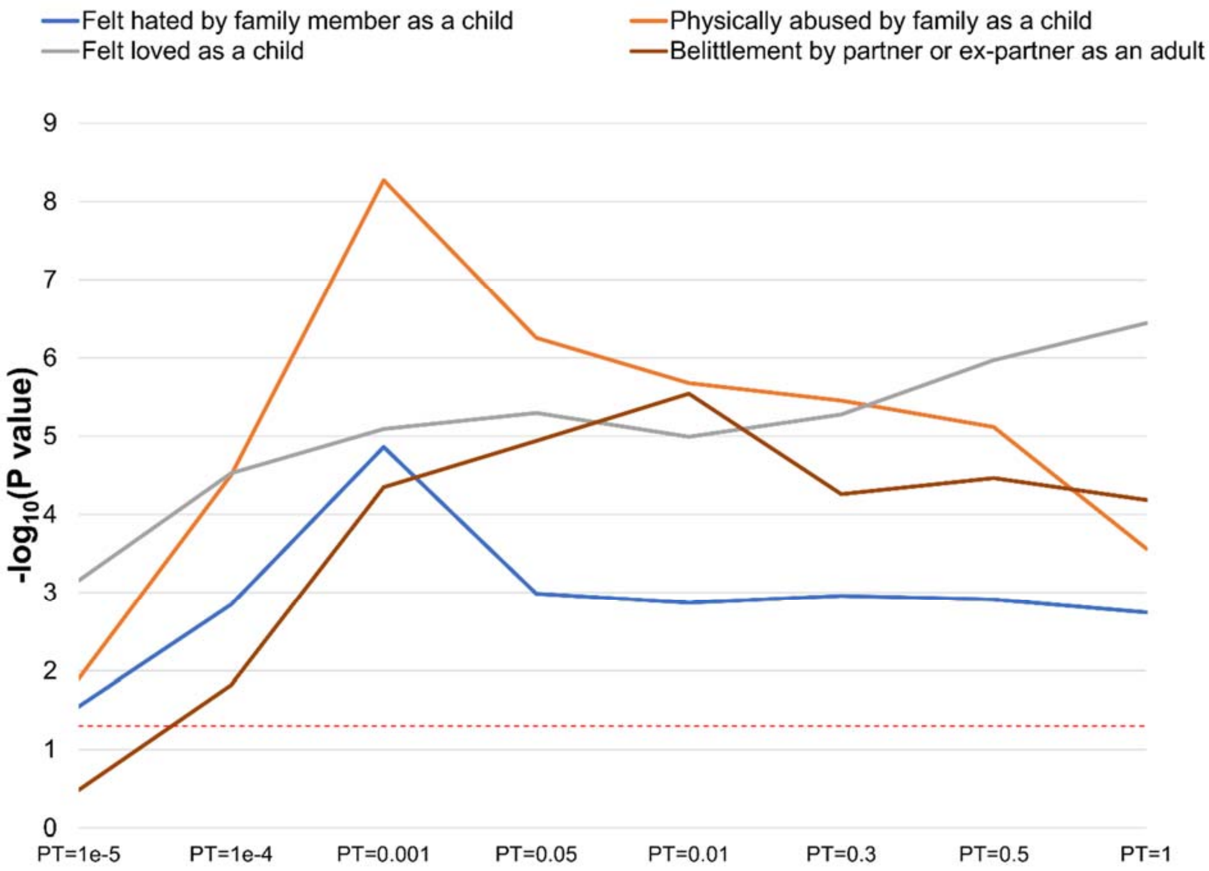

Red dotted line corresponds to nominal significance (p<0.05).

© 2019 Polimanti R et al. *JAMA Network Open.*

eFigure 12: Multivariable Mendelian Randomization Analysis Considering the Effects of Physically Abused by Family as a Child and Belittlement by Partner or Ex-Partner as an Adult on PTSD

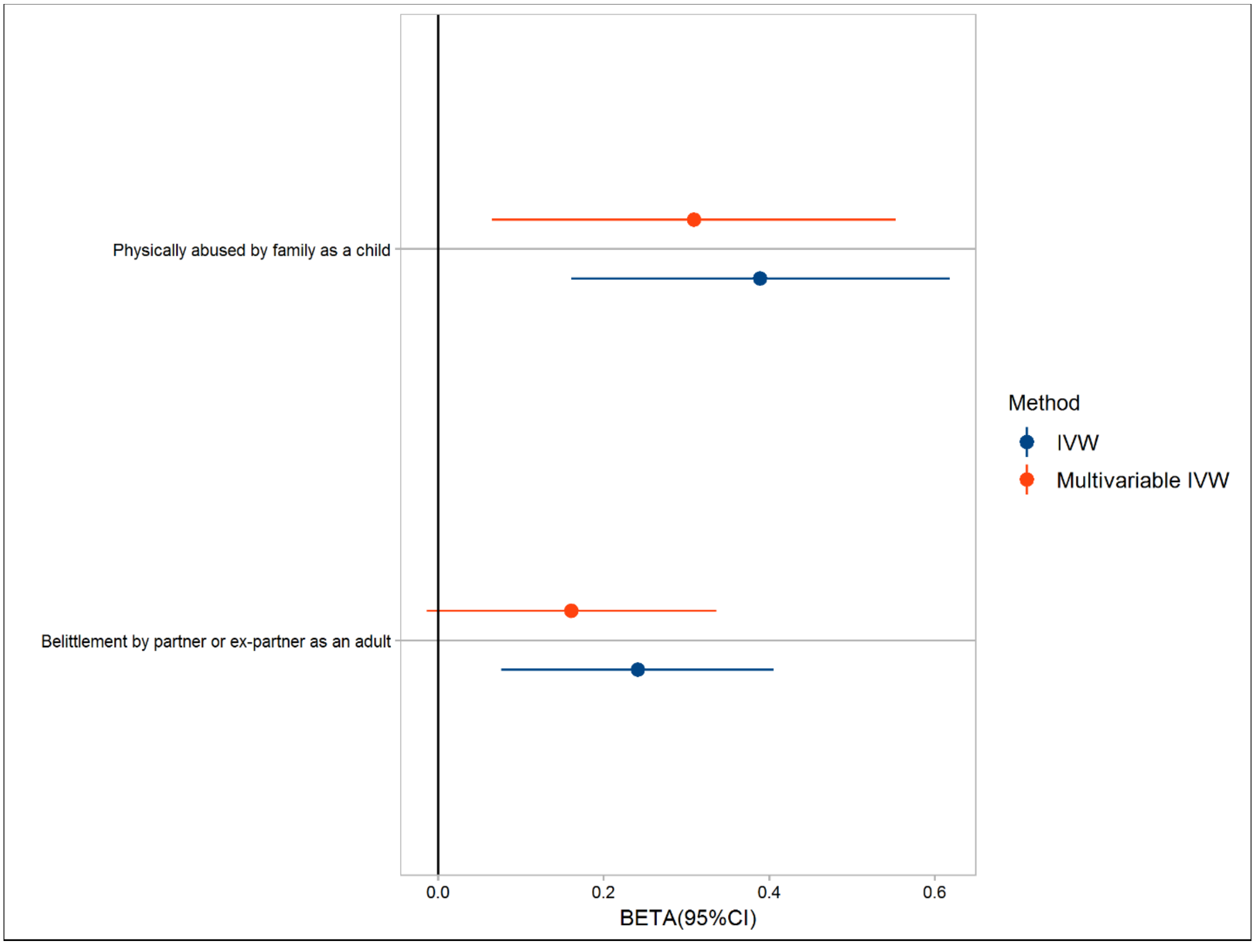

© 2019 Polimanti R et al. *JAMA Network Open.*
